# Novel insights into the aetiology of granulomatosis with polyangiitis—a case–control study using the Clinical Practice Research Datalink

**DOI:** 10.1093/rheumatology/kex512

**Published:** 2018-02-26

**Authors:** Fiona A Pearce, Peter C Lanyon, Richard A Watts, Matthew J Grainge, Abhishek Abhishek, Richard B Hubbard

**Affiliations:** 1 Division of Epidemiology and Public Health, University of Nottingham; 2 Department of Rheumatology, Nottingham University Hospitals NHS Trust, Nottingham; 3 Division of Rheumatology, Orthopaedics and Dermatology, University of Nottingham, Nottingham; 4 Department of Rheumatology, Ipswich Hospital, Ipswich, UK

**Keywords:** anti-neutrophil cytoplasmic antibody-associated vasculitis, granulomatosis with polyangiitis, risk factors, aetiology, vasculitis

## Abstract

**Objectives:**

We aimed to provide insights into the aetiology of granulomatosis with polyangiitis (GPA), by conducting a large case–control study using a general population-based, prospectively collected database of healthcare records.

**Methods:**

We compared all incident cases of GPA in the Clinical Practice Research Datalink 1990–2014, with up to 10 age-, sex- and general practice-matched controls. We identified potential risk factors, recorded numbers of cases and controls exposed to each, and calculated odds ratios (ORs) using conditional logistic regression. Our main analysis excluded data recorded during 1 year before diagnosis, to prevent early symptoms being mistaken for risk factors.

**Results:**

We identified 757 people with GPA and matched 7546 controls. People with GPA were five times more likely to have a previous diagnosis of bronchiectasis (OR = 5.1, 95% CI: 2.7, 9.4; *P* < 0.0001), and these effects remained stable in diagnoses recorded >5 years prior to diagnosis. People with GPA were two to three times more likely than controls to have previous diagnoses of autoimmune diseases or chronic renal impairment, and these effects also remained stable >5 years prior to diagnosis. People with GPA were more likely to have a diagnosis of pulmonary fibrosis (OR = 5.7, 95% CI: 1.7, 19.5; *P* = 0.01) and sinus infections (OR = 2.7, 95% CI: 1.8, 4.2; *P* < 0.0001) recorded in the 3 years before diagnosis, but not before this. We also found former smoking, some medications and higher socio-economic status significantly, but less strongly, associated.

**Conclusion:**

We found novel long-term associations between GPA and pre-existing bronchiectasis and autoimmune diseases.

**Rheumatology key messages**

- Autoimmune diseases and bronchiectasis are associated with an increased risk of developing granulomatosis with polyangiitis.
- Sinus infections are associated with an increased risk of developing granulomatosis with polyangiitis for 4 years before diagnosis.
- Former smoking is associated with an increased risk of developing granulomatosis with polyangiitis compared with never smoking.

## Introduction

Granulomatosis with polyangiitis (GPA) is a rare multisystem condition of unknown aetiology. Much of what we know about the aetiology of this condition is based on case reports, case series and small hospital-based case–controls studies with retrospective collection of exposures [1]. A large population-based case–control study of aetiology with prospectively collected exposure data maximizes information while limiting selection and recall bias. In this study, we have aimed to compare the frequency of possible aetiological exposures between cases and population-based controls in a large database of prospectively collected primary care data.

## Methods

### Source of data

We used data from the Clinical Practice Research Datalink (CPRD), which is one of the largest databases of longitudinal medical records from UK primary care. It was established in 1987, and now contains anonymized healthcare records from more than 13 million people and represents 8% of the UK population. Patients are broadly representative of the UK general population in terms of age, sex and ethnicity [2]. Records contain diagnostic and clinical information coded using Read codes [3]. We followed the CPRD’s recommendations for selecting research-quality patient records and periods of quality data recording by including people contributing acceptable quality data in up-to-standard practices. We used data from all 684 general practices contributing data up to January 2015.

### Study participants and study design

We included all incident cases of GPA diagnosed between 1 January 1990 and 31 December 2014, identified as previously described [4]. Briefly, cases were included if they had a diagnosis of GPA coded in the CPRD or linked Hospital Episode Statistics (HES) [5] records; for these people, date of diagnosis was assumed to be the first recorded code in the CPRD or HES of either GPA or vasculitis; cases had at least 1 year of disease-free registration in the CPRD prior to their first code for GPA or vasculitis to try to exclude prevalent cases. Up to 10 controls were randomly selected for each case, matched on general practice, sex and within 5 years of age. All controls also had to be contributing data to the CPRD continuously for at least 1 year prior to the diagnosis date of their matched case. The diagnosis date of cases and the matching date for each control is called the index date.

### Selection of aetiological risk factors

We collated a list of potential risk factors based on previous research, case reports in the literature and expert physician opinion. We grouped our potential risk factors as follows: vascular diseases (hypertension, cardiovascular disease, gout, type 2 diabetes), autoimmune diseases (type 1 diabetes, under-/over-active thyroid diseases, RA, IBD, coeliac disease, vitiligo and Addison’s disease), chronic lung diseases (chronic obstructive pulmonary disease, alpha-1 antitrypsin deficiency, pulmonary fibrosis and bronchiectasis), renal impairment (acute, chronic), infections (upper respiratory tract, lower respiratory tract, sinus, urinary tract and cellulitis), medications (allopurinol, carbimazole, levothyroxine, propythiouracil, hydralazine, minocycline, SSZ and penicillamine—all of which have been associated with vasculitis), smoking and socio-economic status.

### Code lists

We used the method described by Dave and Petersen [6] to compile lists of Read codes for each clinical diagnosis, or unique CPRD code for each medication. We searched the description fields of the Read code dictionary and Product dictionary supplied by the CPRD using a list of keywords and synonyms and excluding irrelevant codes. Where possible the resulting code lists were cross-referenced with published code lists available at an online clinical codes repository [7]. Full lists of codes are available are provided as supplementary data, available at *Rheumatology* online.

We extracted smoking records from the GP records, and categorized smoking status as never, former, current and unknown. We used the last record before the index date, and repeated the analysis using the last record prior to 1 year before the index date.

We used the Index of Multiple Deprivation 2010 (IMD-10), provided by the Office for National Statistics via a CPRD-linkage agreement, as a surrogate for socio-economic status. This is required because socio-economic data are poorly recorded in the CPRD itself because they do not relate directly to patient care [8]. We categorized IMD-10 into quintiles where quintile 1 is the most deprived, and quintile 5 the least deprived.

### Statistical analysis

For the candidate risk factors that were chronic conditions, we recorded the numbers of cases and controls that had at least one coded record for each of the candidate risk factors prior to their index date. We then repeated the analyses excluding new diagnoses or newly prescribed medications 1 year before the index date to explore whether early symptoms of GPA might be misdiagnosed as other illnesses or result in new prescriptions. For chronic conditions that were significantly associated before 1 year before the index date, we then repeated this four more times, excluding new records in the 2, 3, 4 and 5 years before diagnosis to distinguish long-term risk factors from late-emerging risk factors. For smoking and IMD-10, which are chronic exposures but categorical variables, we recorded the numbers of cases and controls who had each of the categories of exposure. For infections and acute renal impairment, which are acute, resolving and possibly recurring episodes, we recorded the number of cases and controls that had at least one coded record for each type in time periods of 1, 0–1 up to 4–5 years before diagnosis.

We calculated odds ratios (ORs) comparing risk factors in cases compared with controls using conditional logistic regression. Because there are records of delays, often up to a year, between symptom onset and diagnosis of GPA [9], our main analysis excluded data from the year before diagnosis. Statistical analysis was performed using STATA version 14 (Statacorp, College Station, TX, USA).

### Reporting guidelines

This study is reported following the REporting of studies Conducted using Observational Routinely-collected health Data (RECORD) guidelines [10], which are an extension of the STrengthening the Reporting of OBservational studies in Epidemiology (STROBE) statement [11], designed specifically for reporting studies conducted using observational routinely collected healthcare data.

### Ethical approval

Independent Scientific Advisory Committee for Medicines & Healthcare Products Regulatory Agency (MHRA) database research approval was obtained for this study on 21 July 2015 (protocol 15_150 R). Patient consent was not required because no patient-identifiable information was used.

## Results

We identified 757 cases of GPA and matched 7546 controls. The baseline characteristics of cases and controls were similar: median age (interquartile range) at the index date was 61 (50–70) years in cases and 61 (50–71) years in controls, and 44.7% of both cases and controls were female.

### Chronic conditions

**Fig. 1:**
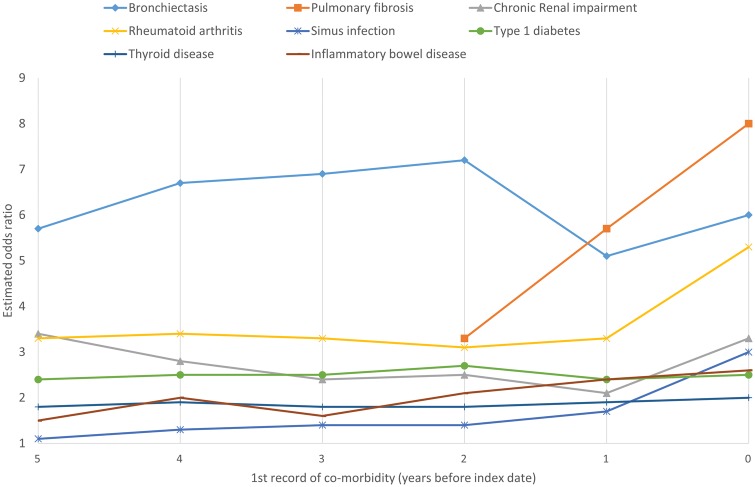
Chronic conditions: association with developing granulomatosis with polyangiitis

Pulmonary fibrosis and bronchiectasis were most strongly associated with GPA (Table 1). In our main results (excluding new diagnoses in the year before their index date), people with GPA were 5.7 times more likely than controls to have a past history of pulmonary fibrosis (OR = 5.7, 95% CI: 1.7, 19.5; *P* = 0.01) and 5.1 times more likely to have bronchiectasis (OR = 5.1, 95% CI: 2.7, 9.4; *P* < 0.0001). Chronic renal impairment (OR = 2.1, 95% CI: 1.5, 3.1; *P* = 0.0001), type 1 diabetes (OR = 2.4, 95% CI: 1.2, 4.6; *P* = 0.02), under-/over-active thyroid diseases (OR = 1.9, 95% CI: 1.4, 2.5; *P* < 0.0001), RA (OR = 3.3, 95% CI: 2.0, 5.3; *P* < 0.0001) and IBD (OR = 2.4, 95% CI: 1.4, 4.2; *P* = 0.004) were all associated with diagnosis of GPA. The associations were similar, but slightly stronger, when new diagnoses during the year before the index date were included. Looking back to 5 years before the index date (Fig. 1 and supplementary Table S1, available at *Rheumatology* online), the risk conferred by bronchiectasis was stable (5 years prior: OR = 5.7, 95% CI: 2.0, 16.1; *P* = 0.003) but the first cases with pulmonary fibrosis appeared 2–3 years before the index date (>2 years prior: OR = 3.3, 95% CI: 0.3, 32.0; *P* = 0.4), and the risk rose rapidly over the 2 years before the index date. The risks conferred by chronic renal failure, RA, type 1 diabetes, thyroid disease and IBD remained stable over the period 1–5 years before the index date, but the ORs tended to increase in the year prior to diagnosis.

**Table 1:** Chronic conditions and their association with developing granulomatosis with polyangiitis

Diagnosis	Ever before the index date				Main analysis: excluding the last year before index date			
	Cases, *n* (%)	Controls, *n* (%)	Odds ratio (95% CI)	*P*-value	Cases, *n* (%)	Controls, *n* (%)	Odds ratio (95% CI)	*P*-value
Vascular								
CVD	74 (9.8)	707 (9.4)	1.1 (0.8, 1.4)	0.7	67 (8.9)	660 (8.8)	1.0 (0.8, 1.3)	0.9
Hypertension	226 (29.9)	2088 (27.7)	1.1 (0.9, 1.4)	0.2	205 (27.1)	1935 (25.6)	1.1 (0.9, 1.3)	0.3
Chronic renal impairment	77 (10.2)	325 (4.3)	3.3 (2.5, 4.6)	<0.0001	49 (6.5)	277 (3.7)	2.1 (1.5, 3.1)	0.0001
Gout	38 (5.0)	284 (3.8)	1.4 (1.0, 2.0)	0.09	33 (4.4)	268 (3.6)	1.2 (0.9, 1.8)	0.3
Type 2 diabetes	56 (7.4)	555 (7.4)	1.0 (0.8, 1.3)	0.96	45 (5.9)	496 (6.6)	0.9 (0.6, 1.2)	0.5
Autoimmune								
Type 1 diabetes	12 (1.6)	48 (0.6)	2.5 (1.3, 4.8)	0.009	11 (1.5)	47 (0.6)	2.4 (1.2, 4.6)	0.02
Thyroid disease	83 (11.0)	453 (6.0)	2.0 (1.6, 2.6)	<0.0001	74 (9.8)	429 (5.7)	1.9 (1.4, 2.5)	<0.0001
RA	40 (5.3)	80 (1.1)	5.3 (3.6, 7.8)	<0.0001	23 (3.0)	72 (1.0)	3.3 (2.0, 5.3)	<0.0001
IBD	18 (2.4)	69 (0.9)	2.6 (1.6, 4.4)	0.001	16 (2.1)	66 (0.9)	2.4 (1.4, 4.2)	0.004
Coeliac disease	2 (0.3)	19 (0.3)	1.1 (0.2, 4.5)	0.9	2 (0.3)	18 (0.3)	1.1 (0.3, 4.8)	0.9
Vitiligo	1 (0.1)	15 (0.2)	0.7 (0.1, 5.1)	0.7	1 (0.1)	14 (0.2)	0.7 (0.1, 5.5)	0.7
Addison’s disease	0	1 (0.01)						
Chronic lung disease								
COPD	41 (5.4)	273 (3.6)	1.5 (1.1, 2.2)	0.02	29 (3.8)	237 (3.2)	1.2 (0.8, 1.8)	0.3
Alpha-1 ATD	0	1 (0.01)						
Pulmonary fibrosis	8 (1.1)	10 (0.1)	8.0 (3.2, 20.3)	0.0001	4 (0.5)	7 (0.1)	5.7 (1.7, 19.5)	0.01
Bronchiectasis	20 (2.6)	34 (0.5)	6.0 (3.4, 10.6)	<0.0001	15 (2.0)	30 (0.4)	5.1 (2.7, 9.4)	<0.0001

CVD: cardiovascular disease; COPD: chronic obstructive pulmonary disease; alpha-1 ATD: alpha-1 antitrypsin deficiency.

### Acute renal impairment

Acute renal impairment occurred in very few cases (*n* = 8) or controls (*n* = 11) >1 year prior to the index date. Acute renal impairment was recorded more often in cases compared with controls throughout 5 years before diagnosis; however, the results were not statistically significant (Table 2).

**Table 2:** Acute, resolving illnesses and their association with developing granulomatosis with polyangiitis

Years before index date	Cases, *n* (%)	Controls, *n* (%)	Odds ratio (95% CI)	*P*-value
Acute renal impairment				
0–1	35 (4.6)	2 (0.03)	175.0 (42.1, 727.6)	<0.0001
1–2	2 (0.3)	5 (0.07)	4.8 (0.9, 26.4)	0.1
2–3	1 (0.2)	3 (0.05)	4.8 (0.4, 53.3)	0.3
3–4	0	3 (0.05)	–	–
4–5	5 (0.1)	0	–	–
Sinus infection				
0–1	110 (14.5)	116 (1.5)	11.2 (8.5, 14.9)	<0.0001
1–2	28 (4.1)	111 (1.6)	2.7 (1.8, 4.2)	<0.0001
2–3	20 (3.2)	86 (1.4)	2.5 (1.5, 4.1)	0.001
3–4	16 (2.8)	86 (1.5)	1.9 (1.1, 3.2)	0.04
4–5	12 (2.3)	86 (1.7)	1.4 (0.7, 2.6)	0.3
Upper respiratory tract infection				
0–1	111 (14.7)	419 (5.6)	3.1 (2.4, 3.9)	<0.0001
1–2	64 (9.5)	397 (5.8)	1.7 (1.3, 2.3)	0.0004
2–3	37 (6.0)	385 (6.2)	1.0 (0.7, 1.4)	0.9
3–4	36 (6.4)	323 (5.7)	1.1 (0, 8, 1.6)	0.6
4–5	43 (8.3)	318 (6.3)	1.3 (0.9, 1.9)	0.1
Lower respiratory tract infection				
0–1	104 (13.7)	331 (4.4)	3.7 (2.9, 4.7)	<0.0001
1–2	39 (5.8)	284 (4.2)	1.4 (1.0, 2.1)	0.05
2–3	23 (3.7)	234 (3.7)	1.0 (0.6, 1.5)	0.9
3–4	28 (5.0)	209 (3.7)	1.4 (0.9, 2.1)	0.1
4–5	17 (3.3)	189 (3.7)	0.9 (0.5, 1.5)	0.7
Urinary tract infection				
0–1	35 (4.6)	204 (2.7)	1.8 (1.2, 2.6)	0.004
1–2	20 (3.0)	201 (2.9)	1.0 (0.6, 1.6)	1.0
2–3	16 (2.6)	163 (2.6)	1.0 (0.6, 1.7)	1.0
3–4	15 (2.7)	150 (2.7)	1.0 (0.6, 1.7)	1.0
4–5	14 (2.7)	117 (2.3)	1.1 (0.6, 1.9)	0.8
Cellulitis				
0–1	29 (3.8)	98 (1.3)	3.1 (2.0, 4.7)	<0.001
1–2	8 (1.2)	97 (1.4)	0.9 (0.4, 1.8)	0.7
2–3	9 (1.5)	67 (1.1)	1.4 (0.7, 2.9)	0.3
3–4	5 (0.9)	69 (1.2)	0.7 (0.3, 1.8)	0.4
4–5	8 (1.5)	54 (1.1)	1.4 (0.7, 2.9)	0.4

### Infections

Sinus infection was the infection most strongly associated with developing GPA, with an OR of 2.7 (95% CI: 1.8, 4.2; *P* < 0.0001) 1–2 years prior to the index date (Table 2). The ORs remained elevated until 4 years before the index date. Upper and lower respiratory tract infections were also less strongly but significantly associated during 1–2 years prior to the index date (OR = 1.7, 95% CI: 1.3, 2.3; *P* = 0.0004; and OR = 1.4, 95% CI: 1.0, 2.1; *P* = 0.05, respectively), but not before this time. There was no association between urinary tract infection or cellulitis and developing GPA.

### Smoking

Former smoking was associated with an increased risk of developing GPA compared with never smokers (OR = 1.5, 95% CI: 1.2, 1.8; *P* < 0.001) (Table 3). Current smoking was marginally associated with a decreased risk of developing GPA compared with never smokers (OR = 0.8, 95% CI: 0.7, 1.0; *P* = 0.077).

**Table 3:** Smoking and its association with developing granulomatosis with polyangiitis

Diagnosis	Last record prior to the index date				Last record prior to 1 year before the index date			
	Cases, *n* (%)	Controls, *n* (%)	Odds ratio (95% CI)	*P*-value	Cases, *n* (%)	Controls, *n* (%)	Odds ratio (95% CI)	*P*-value
Smoking status								
Never	349 (46.1)	33505 (46.5)	1		336 (44.4)	3, 415 (45.3)	1	
Former	172 (22.7)	1241 (16.5)	1.5 (1.2, 1.8)	<0.001	149 (19.7)	1, 074 (14.2)	1.5 (1.2, 1.8)	<0.001
Current	172 (22.7)	2052 (27.2)	0.8 (0.7, 1.0)	0.037	186 (24.6)	2, 177 (28.9)	0.8 (0.7, 1.0)	0.077
Unknown	64 (8.5)	744 (9.9)	0.8 (0.6, 1.1)	0.12	86 (11.4)	880 (11.7)	0.9 (0.7, 1.3)	0.68

### Medications

SSZ was the medication most strongly associated with developing GPA, with an OR 1 year prior to the index date of 3.0 (95% CI: 1.4, 6.4; *P* = 0.01), and an OR 5 years prior of 2.5 (95% CI: 0.9, 6.8; *P* = 0.1) (Table 4; supplementary Fig. S1 and Table S2, available at *Rheumatology* online). The estimated risk conferred by the medications used for thyroid disease were around two and remained stable throughout the 5 years prior to the index date. Allopurinol (OR = 1.8, 95% CI: 1.2, 2.8; *P* = 0.01) was also associated with developing GPA. Penicillamine, propylthiouracil and hydralazine were prescribed too rarely for us to be able to comment with confidence on any possible association.

**Table 4:** Medications and their association with developing granulomatosis with polyangiitis

Drug	Ever before the index date				Excluding the last year before the index date			
	Cases, *n* (%)	Controls, *n* (%)	Odds ratio (95% CI)	*P*-value	Cases, *n* (%)	Controls, *n* (%)	Odds ratio (95% CI)	*P*-value
Allopurinol	30 (4.0)	163 (2.2)	1.9 (1.3, 2.9)	0.0032	26 (3.4)	147 (2.0)	1.8 (1.2, 2.8)	0.01
Carbimazole	9 (1.2)	46 (0.6)	2.0 (1.0, 4.1)	0.08	8 (1.1)	43 (0.6)	1.9 (0.9, 4.1)	0.13
Levothyroxine	71 (9.4)	353 (4.7)	2.2 (1.7, 2.9)	<0.0001	62 (8.2)	332 (4.4)	2.0 (1.5, 2.7)	<0.0001
Propylthiouracil	1 (0.1)	7 (0.1)	1.4 (0.2, 11.5)	0.76	1 (0.1)	5 (0.1)	2.0 (0.2, 16.8)	0.57
Hydralazine	2 (0.3)	9 (0.1)	2.3 (0.5, 10.8)	0.34	2 (0.3)	8 (0.1)	2.6 (0.5, 12.5)	0.29
Minocycline	17 (2.3)	94 (1.3)	1.8 (1.1, 3.1)	0.03	14 (1.9)	90 (1.2)	1.6 (0.9, 2.8)	0.1
SSZ	14 (1.9)	31 (0.4)	4.6 (2.4, 8.6)	<0.0001	9 (1.2)	30 (0.4)	3.0 (1.4, 6.4)	0.01
Penicillamine	4 (0.5)	10 (0.1)	4.0 (1.3, 12.8)	0.04	4 (0.5)	10 (0.1)	4.0 (1.3, 12.8)	0.04

### Socio-economic status

IMD-10 data were available for the 5573 (67.1%) of our study population who were registered at GP practices in England. There was a weak association between decreasing socio-economic deprivation and increased risk of GPA (Table 5). The least deprived IMD-10 quintile were estimated to be 30% more likely than the most deprived to develop GPA (OR = 1.3, 95% CI: 0.9, 1.9; *P*-trend 0.02).

**Table 5:** Socio-economic status and its association with developing granulomatosis with polyangiitis

IMD-10 quintile	Ever before the index date				
	Cases, *n* (%)	Controls, *n* (%)	Odds ratio (95% CI)— categorical variable	Odds ratio (95% CI)— continuous variable	*P*-value^a^
Quintile 1 (most deprived)	113 (22.2)	1220 (24.1)	1	1.1 (1.0, 1.2)	0.02
Quintile 2	114 (22.4)	1303 (25.7)	0.96 (0.7, 1.3)		
Quintile 3	114 (22.4)	1041 (20.6)	1.3 (0.9, 1.8)		
Quintile 4	102 (20.0)	854 (16.9)	1.5 (1.0, 2.1)		
Quintile 5 (least deprived)	66 (13.0)	646 (12.8)	1.3 (0.9, 1.9)		

a *P*-value for trend from the likelihood ratio test. IMD-10: Index of Multiple Deprivation 2010.

## Discussion

### Main results

To our knowledge this is the largest case–control study of the aetiology of GPA. We found that people with GPA were two to three times more likely than population-based controls to have a previous diagnosis of an autoimmune disease or chronic renal impairment, and these effects remained stable when we looked at diagnoses recorded >5 years prior to diagnosis. People with GPA were also more likely to have pulmonary fibrosis and sinus infections at diagnosis, but these effects appeared in the 3 years prior to diagnosis, and were either late risk factors or early symptoms of GPA. Intriguingly, we found that people with GPA were five times more likely than population-based controls to have a previous diagnosis of bronchiectasis, and these effects remained stable when we looked at diagnoses recorded >5 years prior to diagnosis. To our knowledge, an association between bronchiectasis and GPA has not previously been reported.

### Strengths and limitations

The main strengths of our study are the large number of people in the study population, and the long follow-up period. In addition, the population-based nature of our case ascertainment should include people who are representative of the full spectrum of people with GPA, in contrast to the selected cohorts included in studies in tertiary referral centres or clinical trials [12]. Using prospectively collected data from an electronic health record limits recall bias, which is a major consideration in studies of aetiology. The main limitations of our study include lack of power for the risk factors that occur infrequently in controls, and the possibility of misclassification of diagnoses and other exposures in the CPRD. Previous studies have demonstrated the high (90%) positive predictive value of the recording of a diagnosis of GPA [13], and similarly high validity of records of other chronic medical diagnoses, infections and prescription records [14–16] in the CPRD. Unfortunately, it is no longer possible to check these in our sample using anonymized hospital correspondence from the CPRD due to a tightening of their procedures to protect confidentiality. The quality of smoking data is enhanced by the Quality and Outcomes framework, which provides an incentive payment to English GPs for recording key data items. We have ensured good quality of socio-economic data by using the IMD-10, the official measure of relative deprivation for neighbourhoods in England; however, these data are only available for people who consent to this linkage and who are registered at GP practices in England, comprising 67% of our dataset. Despite this theoretical limitation, we found a small but statistically significant increased risk of developing GPA among the least deprived people compared with the most deprived group.

### How our study fits in with the other literature

Bronchiectasis was the most strongly associated long-term risk factor in our study. Bronchiectasis is already known to be associated with other autoimmune diseases, particularly RA [17, 18]. Bronchiectasis has previously been reported in two retrospective single centre studies from France and Japan to be associated with microscopic polyangiitis and anti-myeloperoxidase ANCA-associated vasculitis (AAV) [19, 20], and there are reports of people with bronchiectasis and an atypical p-ANCA directed against bactericidal/permeability-increasing protein ANCA, often after *Pseudomonas aeruginosa* infection of the lungs [21–23]. This is the first study we are aware of reporting a long-term association between bronchiectasis and GPA. Three possible reasons for the association exist: that bronchiectasis contributes to causing GPA, that they share aetiological factors or that they are both manifestations of the same disease spectrum. It is particularly intriguing because the aetiology of bronchiectasis is unknown, and there are some case reports of bronchiectasis resolving with immunosuppression [24, 25].

The association with pulmonary fibrosis is interesting, because it has previously been associated with microscopic polyangiitis, but not specifically with GPA. This association in the 1–3 years before diagnosis could be due to any of the same three reasons as bronchiectasis, but because it is a late association it is more likely to be a manifestation of the same disease. The more common chronic lung disease, chronic obstructive pulmonary disease (including emphysema), on the other hand, did not show an association with developing GPA, which is possibly due to a protective effect of smoking. Alpha-1 anti-trypsin deficiency, which has previously been associated [26], was too rare in our cohort for us to comment.

We found that type 1 diabetes, under-/over-active thyroid diseases, RA and IBD occurred two to three times more commonly in cases than in the general population controls. Coeliac disease, vitiligo and Addison’s disease occurred in zero to two cases each only, so we have insufficient power to comment in these conditions. Autoimmune diseases are well-recognized to cluster in individuals and families [27–29]; however, this is one of the first studies we are aware of reporting the association of GPA with several other autoimmune diseases. Lionaki *et al.* conducted a case–control study of 158 cases of AAV, and found a history of thyroid disease to be more common in cases than in age- and sex-matched controls (OR = 3.7, 95% CI: 1.5, 9.2; *P* = 0.005) [30]. Other evidence comes from case series: there have been reports of 6 cases where AAV occurred after a diagnosis of RA [31], 7 cases of co-existence of primary SS and AAV [32] and 35 cases of co-existence of scleroderma and AAV [33]. It seems likely that the finding of clustering of autoimmune diseases in individuals [29] is contributed to by shared genetic susceptibility loci at immune regulatory genes (HLA [34], CTLA-4 [35, 36], PTPN22 [37] and CD40 [38]) [39, 40]. Other evidence pointing to shared mechanisms of immune dysregulation in autoimmune conditions include that atypical ANCAs also occur frequently in IBD, RA and thyroid disease [41].

Our study did not provide evidence of infections as long-term risk factors for GPA, with the exception of sinus infections. Sinus infections occurred more than twice as commonly in people with GPA compared with controls up to 4 years before diagnosis. All types of infection were significantly associated only in the last year before the index date, and it may be that the early symptoms of GPA were mistaken for infections, or that the concept of infections acting as a stochastic event that triggers the sudden flare of GPA may be true. In our dataset we were unable to specifically identify *Staphylococcus aureus* infections, so we are unable to comment on the previously reported association [42].

Chronic renal impairment, and possibly acute renal impairment, were also, surprisingly, more frequently recorded before 1 year prior to diagnosis in people with GPA compared with controls. This association has not, to our knowledge, been reported before. For chronic renal impairment, the size of the effect remained significant at least 5 years before the index date. Other diseases associated with vascular damage, such as hypertension, type 2 diabetes, cardio-vascular disease and gout, were not increased in people with GPA, although they have been previously reported as being more common after diagnosis [43]. It therefore does not seem that vascular damage triggers GPA, but raises the possibility of minor flares of renal vasculitis causing renal damage but not fulminant vasculitis, over at least 5 years prior to diagnosis in up to 3–5% of cases.

Several drugs have previously been associated with GPA [1]. In our study we have attempted to quantify the effect of previously implicated drugs compared with the effect of having the prescribing indication, by presenting, for example, the ORs for gout and allopurinol. Allopurinol appears to confer a risk of developing GPA that is greater than the risk conferred by its indication. However, SSZ conferred a similar risk to that of RA itself, and carbimazole and levothyroxine conferred a risk similar to thyroid disease itself. It should be noted that the number of cases prescribed penicillamine, propylthiouracil and hydralazine were so small that these results should be interpreted cautiously. We were unable to include thioridazine as there were no cases, and levamisole (used to adulterate cocaine) because we were unable to quantify its use.

Former smoking was associated with developing GPA, which is a further novel finding of our study. We also found that current smoking was possibly slightly protective, as has been shown previously in a small study comparing the smoking habits at diagnosis of AAV in 197 German people, with the smoking prevalence in the German population [44]. This could be due to the immunosuppressive effect of smoking, which is caused mainly by the inhalation of nicotine [45]. Few studies have been reported of the effects on the immune system of stopping smoking. A similar association with former smoking has been found in sarcoid [46], which is also another granulomatous disease. However, this line of thought does not extend to IBD, where Crohn’s disease, which is granulomatous, is more frequent in smokers whereas in ulcerative colitis, which is not granulomatous, smoking is protective [47].

Our finding that the incidence of GPA is weakly associated with higher socio-economic status is almost identical to the finding of a previous study from New Zealand of 195 cases of ANCA-associated vasculitis ascertained from coding of hospital inpatient discharges, which found a statistically significant lower incidence of granulomatosis with polyangiitis among the most deprived 2 socio-economic status deciles compared to all the other deciles [48].

### Clinical implications and conclusion

Our study found a novel association between pre-existing bronchiectasis and the development of GPA, and confirmed previously described associations with autoimmune diseases, renal impairment, pulmonary fibrosis, sinus infection and previous smoking.

## Supplementary Material

Supplementary Data
