# Novel insights into the aetiology of granulomatosis with polyangiitis—a case–control study using the Clinical Practice Research Datalink

**DOI:** 10.1093/rheumatology/key249

**Published:** 2018-07-27

**Authors:** Fiona A Pearce, Peter C Lanyon, Richard A Watts, Matthew J Grainge, Abhishek Abhishek, Richard B Hubbard

**Affiliations:** 1 Division of Epidemiology and Public Health, University of Nottingham, Nottingham, UK; 2 Department of Rheumatology, Nottingham University Hospitals NHS Trust, Nottingham, UK; 3 Division of Rheumatology, Orthopaedics and Dermatology, University of Nottingham, Nottingham, UK; 4 Department of Rheumatology, Ipswich Hospital, Ipswich, UK

*Rheumatology* 2018;57:1002–1010. doi: 10.1093/rheumatology/kex512 This article was amended to include the research grant number. Additionally, a correction was made to the discussion section to acknowledge the findings of a previous study in this area.

