# Supplementary material for: Novel insights into the aetiology of granulomatosis with polyangiitis—a case–control study using the Clinical Practice Research Datalink
Source: Rheumatology (Oxford). 2018 Feb 26;57(6):1002–10. doi: 10.1093/rheumatology/kex512 (PMC5965083; doi:10.1093/rheumatology/kex512)
Supplement: Supplementary Data [file kex512_rhe-17-1276-file004.docx]

##### **SUPPLEMENTARY DATA**

**Supplementary table S1: Chronic conditions and their association with developing granulomatosis with polyangiitis**

| **Bronchiectasis** | | | | | | | | |
| --- | --- | --- | --- | --- | --- | --- | --- | --- |
| **Years before index date** | **Cases, n (%)** | | **Controls, n (%)** | | **Odds ratio (95% CI)** | | **P value** | |
| **Ever** | 20 (2.6) | | 34 (0.5) | | 6.0 (3.4-10.6) | | <0.0001 | |
| **>1** | 15 (2.0) | | 30 (0.4 ) | | 5.1 (2.7-9.4) | | <0.0001 | |
| **>2** | 14 (2.1) | | 21 (0.3) | | 7.2 (3.6-14.3) | | <0.0001 | |
| **>3** | 11 (1.8) | | 16 (0.3) | | 6.9 (3.1-15.1) | | <0.0001 | |
| **>4** | 9 (1.6) | | 13 (0.2) | | 6.7 (2.8-15.9) | | 0.0001 | |
| **>5** | 6 (1.2) | | 10 (0.2) | | 5.7 (2.0-16.1) | | 0.003 | |
| **Pulmonary Fibrosis** | | | | | | | | |
| **Years before index date** | | **Cases, n (%)** | | **Controls, n (%)** | | **Odds ratio (95% CI)** | | **P value** |
| **Ever** | | 8 (1.1) | | 10 (0.1) | | 8.0 (3.2-20.3) | | 0.0001 |
| **>1** | | 4 (0.5) | | 7 (0.1) | | 5.7 (1.7-19.5) | | 0.01 |
| **>2** | | 1 (0.2) | | 4 (0.1) | | 3.3 (0.3-32.0) | | 0.4 |
| **>3** | | 0 (0) | | 3 (0.1) | | - | | - |
| **>4** | | 0 (0) | | 3 (0.1) | | - | | - |
| **>5** | | 0 (0) | | 3 (0.1) | | - | | - |
| **Rheumatoid arthritis** | | | | | | | | |
| **Years before index date** | **Cases, n (%)** | | **Controls, n (%)** | | **Odds ratio (95% CI)** | | **P value** | |
| **Ever** | 40 (5.35) | | 80 (1.1) | | 5.3 (3.6-7.8) | | <0.0001 | |
| **>1** | 23 (3.0) | | 72 (1.0) | | 3.3 (2.0-5.3) | | <0.0001 | |
| **>2** | 18 (2.7) | | 60 (0.9) | | 3.1 (1.8-5.4) | | 0.0002 | |
| **>3** | 16 (2.6) | | 50 (0.8) | | 3.3 (1.9-6.0) | | 0.0003 | |
| **>4** | 15 (2.7) | | 43 (0.8) | | 3.4 (1.8-6.2) | | 0.0004 | |
| **>5** | 13 (2.5) | | 38 (0.8) | | 3.3 (1.7-6.3) | | 0.001 | |
| **Inflammatory Bowel Disease** | | | | | | | | |
| **Years before index date** | | **Cases, n (%)** | | **Controls, n (%)** | | **Odds ratio (95% CI)** | | **P value** |
| **Ever** | | 18 (2.4) | | 69 (0.9) | | 2.6 (1.6-4.4) | | 0.001 |
| **>1** | | 16 (2.1) | | 66 (0.9) | | 2.4 (1.4-4.2) | | 0.004 |
| **>2** | | 13 (1.9) | | 61 (0.9) | | 2.1 (1.2-3.9) | | 0.02 |
| **>3** | | 10 (1.6) | | 59 (0.9) | | 1.6 (0.8-3.2) | | 0.2 |
| **>4** | | 10 (1.8) | | 49 (0.9) | | 2.0 (1.0-4.0) | | 0.07 |
| **>5** | | 6 (1.2) | | 38 (0.8) | | 1.5 (0.6-3.6) | | 0.4 |
| **Type 1 Diabetes** | | | | | | | | |
| **Years before index date** | | **Cases, n (%)** | | **Controls, n (%)** | | **Odds ratio (95% CI)** | | **P value** |
| **Ever** | | 12 (1.6) | | 48 (0.6) | | 2.5 (1.3-4.8) | | 0.009 |
| **>1** | | 11 (1.5) | | 47 (0.6) | | 2.4 (1.2-4.6) | | 0.02 |
| **>2** | | 10 (1.5) | | 40 (0.6) | | 2.7 (1.3-5.4) | | 0.01 |
| **>3** | | 9 (1.5) | | 37 (0.6) | | 2.5 (1.2-5.3) | | 0.03 |
| **>4** | | 9 (1.6) | | 35 (0.6) | | 2.6 (1.2-5.5) | | 0.02 |
| **>5** | | 8 (1.5) | | 33 (0.7) | | 2.4 (1.1-5.2) | | 0.05 |
| **Thyroid disease** | | | | | | | | |
| **Years before index date** | **Cases, n (%)** | | **Controls, n (%)** | | **Odds ratio (95% CI)** | | **P value** | |
| **Ever** | 83 (11.0) | | 453 (6.0) | | 2.0 (1.6-2.6) | | <0.0001 | |
| **>1** | 74 (9.8) | | 429 (5.7) | | 1.9 (1.4-2.5) | | <0.0001 | |
| **>2** | 62 (9.2) | | 375 (5.5) | | 1.8 (1.4-2.5) | | 0.0001 | |
| **>3** | 55 (8.9) | | 333 (5.3) | | 1.8 (1.3-2.5) | | 0.0003 | |
| **>4** | 49 (8.7) | | 288 (5.1) | | 1.9 (1.3-2.6) | | 0.0005 | |
| **>5** | 43 (8.3) | | 248 (4.9) | | 1.8 (1.3-2.6) | | 0.0012 | |
| **Chronic Renal impairment** | | | | | | | | |
| **Years before index date** | **Cases, n (%)** | | **Controls, n (%)** | | **Odds ratio (95% CI)** | | **P value** | |
| **Ever** | 77 (10.2) | | 325 (4.3) | | 3.3 (2.5-4.6) | | <0.0001 | |
| **>1** | 49 (6.5) | | 277 (3.7) | | 2.1 (1.5-3.1) | | 0.0001 | |
| **>2** | 39 (5.8) | | 196 (2.9) | | 2.5 (1.6-3.7) | | <0.0001 | |
| **>3** | 27 (4.4) | | 142 (2.3) | | 2.4 (1.5-3.8) | | 0.0008 | |
| **>4** | 20 (3.5) | | 94 (1.7) | | 2.8 (1.6-5.0) | | 0.0007 | |
| **>5** | 15 (2.9) | | 65 (1.3) | | 3.4 (1.8-6.6) | | 0.0006 | |

Each line of these tables contains data incrementally excluding annual time periods up to 5 years before the index date. Only conditions that were significantly associated at >1 year are included. Only people under active f/u in the CPRD in the time period covered are included, so the number of cases and controls decreases with each year to 519 cases and 5,081 controls with >5 years follow up before the index date.

**Supplementary table S2: Medications and their association with developing granulomatosis with polyangiitis**

| **Allopurinol** | | | | |
| --- | --- | --- | --- | --- |
| **Years before index date** | **Cases, n (%)** | **Controls, n (%)** | **Odds ratio (95% CI)** | **P value** |
| **Ever** | 30 (4.0) | 163 (2.2) | 1.9 (1.3-2.9) | 0.0032 |
| **>1** | 26 (3.4) | 147 (2.0) | 1.8 (1.2-2.8) | 0.01 |
| **>2** | 23 (3.4) | 124 (1.8) | 1.9 (1.2-3.0) | 0.01 |
| **>3** | 21 (3.4) | 109 (1.7%0 | 2.0 (1.2-3.2) | 0.01 |
| **>4** | 18 (3.2) | 90 (1.6) | 1.9 (1.1-3.3) | 0.02 |
| **>5** | 14 (2.7) | 72 (1.4) | 1.8 (1.0-3.3) | 0.06 |
| **Levothyroxine** | | | | |
| **Years before index date** | **Cases, n (%)** | **Controls, n (%)** | **Odds ratio (95% CI)** | **P value** |
| **Ever** | 71 (9.4) | 353 (4.7) | 2.2 (1.7-2.9) | <0.0001 |
| **>1** | 62 (8.2) | 332 (4.4) | 2.0 (1.5-2.7) | <0.0001 |
| **>2** | 56 (8.3) | 294 (4.3) | 2.1 (1.6-2.9) | <0.0001 |
| **>3** | 49 (7.9) | 260 (4.2) | 2.1 (1.5-2.9) | <0.0001 |
| **>4** | 44 (7.8) | 216 (3.8) | 2.2 (1.6-3.1) | <0.0001 |
| **>5** | 35 (6.7) | 187 (3.7) | 1.9 (1.3-2.9) | 0.0015 |
| **Sulfasalazine** | | | | |
| **Years before index date** | **Cases, n (%)** | **Controls, n (%)** | **Odds ratio (95% CI)** | **P value** |
| **Ever** | 14 (1.9) | 31 (0.4) | 4.6 (2.4-8.6) | <0.0001 |
| **>1** | 9 (1.2) | 30 (0.4) | 3.0 (1.4-6.4) | 0.01 |
| **>2** | 7 (1.0) | 26 (0.4) | 2.6 (1.1-6.1) | 0.04 |
| **>3** | 7 (1.1) | 23 (0.4) | 2.9 (1.2-6.8) | 0.03 |
| **>4** | 7 (1.2) | 20 (0.4) | 3.0 (1.3-7.3) | 0.02 |
| **>5** | 5 (1.0) | 17 (0.3) | 2.5 (0.9-6.8) | 0.1 |
| **Penicillamine** | | | | |
| **Years before index date** | **Cases, n (%)** | **Controls, n (%)** | **Odds ratio (95% CI)** | **P value** |
| **Ever** | 4 (0.5) | 10 (0.1) | 4.0 (1.3-12.8) | 0.04 |
| **>1** | 4 (0.5) | 10 (0.1) | 4.0 (1.3-12.8) | 0.04 |
| **>2** | 3 (0.4) | 10 (0.2) | 3.3 (0.9-12.1) | 0.11 |
| **>3** | 3 (0.5) | 9 (0.1) | 3.4 (0.9-12.8) | 0.11 |
| **>4** | 3 (0.5) | 9 (0.2) | 3.3 (0.9-12.5) | 0.11 |
| **>5** | 2 (0.4) | 9 (0.2) | 2.0 (0.4-9.7) | 0.4 |

Each line of these tables contains data incrementally excluding annual time periods up to 5 years before the index date. Only medications that were significantly associated at >1 year are included. Only people under active f/u in the CPRD in the time period covered are included, so the number of cases and controls decreases with each year to 519 cases and 5,081 controls with >5 years follow up before the index date.

**Supplementary figure S1. Medications: association with developing GPA**

##### Read codes used in the analysis

| CPRD medcode | Read code | Description |
| --- | --- | --- |
| Granulomatosis with polyangiitis code | | |
| 4810 | G754.00 | Wegener's granulomatosis |
| Hypertension codes | |  |
| 1894 | G201.00 | Benign essential hypertension |
| 63466 | G23..00 | Hypertensive heart and renal disease |
| 20497 | TJC7z00 | Adverse reaction to antihypertensives NOS |
| 7057 | G2z..00 | Hypertensive disease NOS |
| 21660 | TJC7.00 | Adverse reaction to other antihypertensives |
| 30770 | U60C511 | [X] Adverse reaction to other antihypertensives |
| 102458 | Gyu2000 | [X]Other secondary hypertension |
| 24127 | 9OIA.11 | Hypertension monitored |
| 3425 | 662O.00 | On treatment for hypertension |
| 52427 | G211.00 | Benign hypertensive heart disease |
| 13186 | 662P.00 | Hypertension monitoring |
| 97533 | Gyu2100 | [X]Hypertension secondary to other renal disorders |
| 18482 | 662c.00 | Hypertension six month review |
| 99259 | 662q.00 | Trial reduction of antihypertensive therapy |
| 22333 | 8I3N.00 | Hypertension treatment refused |
| 22356 | 1JD..00 | Suspected hypertension |
| 63260 | SLC6z00 | Hypertensive agent poisoning NOS |
| 67232 | G230.00 | Malignant hypertensive heart and renal disease |
| 52127 | G211100 | Benign hypertensive heart disease with CCF |
| 204 | G2...00 | Hypertensive disease |
| 103046 | G210z00 | Malignant hypertensive heart disease NOS |
| 85944 | 7Q01.00 | High cost hypertension drugs |
| 10961 | 9h31.00 | Excepted from hypertension qual indicators: Patient unsuit |
| 15106 | G22z.00 | Hypertensive renal disease NOS |
| 16059 | G24z.00 | Secondary hypertension NOS |
| 21837 | G232.00 | Hypertensive heart&renal dis wth (congestive) heart failure |
| 15377 | G200.00 | Malignant essential hypertension |
| 12680 | 8CR4.00 | Hypertension clinical management plan |
| 71433 | 66b2.00 | Hypertension monitoring not required |
| 351 | G20..11 | High blood pressure |
| 16292 | G21..00 | Hypertensive heart disease |
| 105487 | G26..11 | Severe hypertension |
| 51635 | G241z00 | Secondary benign hypertension NOS |
| 105989 | G26..00 | Severe hypertension (Nat Inst for Health Clinical Ex 2011) |
| 19070 | 662d.00 | Hypertension annual review |
| 43935 | G221.00 | Benign hypertensive renal disease |
| 57288 | G241.00 | Secondary benign hypertension |
| 5433 | F282.00 | Benign intracranial hypertension |
| 10818 | G20z.00 | Essential hypertension NOS |
| 4444 | 662..12 | Hypertension monitoring |
| 799 | G20..00 | Essential hypertension |
| 68659 | G23z.00 | Hypertensive heart and renal disease NOS |
| 28684 | G233.00 | Hypertensive heart and renal disease with renal failure |
| 32423 | G222.00 | Hypertensive renal disease with renal failure |
| 31464 | G21z.00 | Hypertensive heart disease NOS |
| 16565 | 6627.00 | Good hypertension control |
| 63000 | G231.00 | Benign hypertensive heart and renal disease |
| 8732 | G2...11 | BP - hypertensive disease |
| 27511 | 6628.00 | Poor hypertension control |
| 62718 | G21z100 | Hypertensive heart disease NOS with CCF |
| 27525 | 9OI..11 | Hypertension clinic admin. |
| 34281 | 9N4L.00 | DNA - Did not attend hypertension clinic |
| 105274 | G28..00 | Stage 2 hypertension (NICE - Nat Ins for Hth Clin Excl 2011) |
| 7329 | G24..00 | Secondary hypertension |
| 4668 | G22..00 | Hypertensive renal disease |
| 18590 | 662b.00 | Moderate hypertension control |
| 5129 | J623.00 | Portal hypertension |
| 34744 | G244.00 | Hypertension secondary to endocrine disorders |
| 73293 | G240z00 | Secondary malignant hypertension NOS |
| 72668 | G210100 | Malignant hypertensive heart disease with CCF |
| 105480 | G27..00 | Hypertension resistant to drug therapy |
| 101649 | 7Q01y00 | Other specified high cost hypertension drugs |
| 98230 | 67H8.00 | Lifestyle advice regarding hypertension |
| 45149 | 9OI1.00 | Attends hypertension monitor. |
| 106279 | 8IA5.00 | Trial withdrawal of antihypertensive therapy declined |
| 59383 | G240000 | Secondary malignant renovascular hypertension |
| 39649 | G220.00 | Malignant hypertensive renal disease |
| 105371 | G25..00 | Stage 1 hypertension (NICE - Nat Ins for Hth Clin Excl 2011) |
| 31387 | G24z000 | Secondary renovascular hypertension NOS |
| 29310 | G22z.11 | Renal hypertension |
| 95359 | 662r.00 | Trial withdrawal of antihypertensive therapy |
| 72226 | SLC6.00 | Other hypertensive agent poisoning |
| 32976 | 6146200 | Hypertension induced by oral contraceptive pill |
| 10632 | 246M.00 | White coat hypertension |
| 26631 | Q000.00 | Fetus or neonate affected by maternal hypertensive disease |
| 37086 | F404200 | Blind hypertensive eye |
| 36305 | 9OIA.00 | Hypertension monitor.chck done |
| 61166 | G21z000 | Hypertensive heart disease NOS without CCF |
| 5513 | 8HT5.00 | Referral to hypertension clinic |
| 34108 | 9h3..00 | Exception reporting: hypertension quality indicators |
| 4344 | 9N03.00 | Seen in hypertension clinic |
| 83473 | G203.00 | Diastolic hypertension |
| 21826 | 662F.00 | Hypertension treatm. started |
| 21526 | L123500 | Gestational hypertension |
| 10976 | 9h32.00 | Excepted from hypertension qual indicators: Informed dissent |
| 30776 | 6629.00 | Hypertension:follow-up default |
| 102406 | 662P000 | Hypertension 9 month review |
| 6702 | F421300 | Hypertensive retinopathy |
| 27634 | 9N1y200 | Seen in hypertension clinic |
| 31755 | G240.00 | Secondary malignant hypertension |
| 42229 | G24zz00 | Secondary hypertension NOS |
| 25371 | G241000 | Secondary benign renovascular hypertension |
| 31816 | G672.11 | Hypertensive crisis |
| 18057 | 8B26.00 | Antihypertensive therapy |
| 16173 | G21zz00 | Hypertensive heart disease NOS |
| 4372 | G202.00 | Systolic hypertension |
| 105938 | G211z00 | Benign hypertensive heart disease NOS |
| 107704 | G20..12 | Primary hypertension |
| 61660 | G211000 | Benign hypertensive heart disease without CCF |
| 95334 | G210000 | Malignant hypertensive heart disease without CCF |
| 43220 | 9OI2.00 | Refuses hypertension monitor. |
| 18765 | G2y..00 | Other specified hypertensive disease |
| 11056 | 8BL0.00 | Patient on maximal tolerated antihypertensive therapy |
| 5215 | 9OI..00 | Hypertension monitoring admin. |
| 105316 | G25..11 | Stage 1 hypertension |
| 8857 | G21z011 | Cardiomegaly - hypertensive |
| 50157 | G210.00 | Malignant hypertensive heart disease |
| 3712 | G20z.11 | Hypertension NOS |
| 44350 | U60C51A | [X] Adverse reaction to antihypertensives NOS |
| 12948 | 662H.00 | Hypertension treatm.stopped |
| 31341 | G24z100 | Hypertension secondary to drug |
| 69753 | Gyu2.00 | [X]Hypertensive diseases |
| 2666 | 14A2.00 | H/O: hypertension |
| 3979 | G672.00 | Hypertensive encephalopathy |
| 13188 | 662G.00 | Hypertensive treatm.changed |
| 44549 | L128.00 | Pre-exist hypertension compl preg childbirth and puerperium |
| 66567 | L122.00 | Other pre-existing hypertension in preg/childbirth/puerp |
| 72030 | L122100 | Other pre-existing hypertension in preg/childb/puerp - deliv |
| 62432 | L122z00 | Other pre-existing hypertension in preg/childb/puerp NOS |
| 73586 | L122000 | Other pre-existing hypertension in preg/childb/puerp unspec |
| Cardiovascular disease codes | | |
| 10444 | 14A..00 | H/O: cardiovascular disease |
| 35674 | 14A3.00 | H/O: myocardial infarct <60 |
| 40399 | 14A4.00 | H/O: myocardial infarct >60 |
| 6336 | 14A5.00 | H/O: angina pectoris |
| 50372 | 14AH.00 | H/O: Myocardial infarction in last year |
| 57062 | 14AJ.00 | H/O: Angina in last year |
| 45476 | 14AL.00 | H/O: Treatment for ischaemic heart disease |
| 100139 | 14AT.00 | History of myocardial infarction |
| 105216 | 14AW.00 | H/O acute coronary syndrome |
| 103655 | 187..00 | Frequency of angina |
| 101166 | 1I11.00 | Coronary artery disease excluded |
| 30963 | 1J61.00 | Suspected ischaemic heart disease |
| 8246 | 322..00 | ECG: myocardial ischaemia |
| 17820 | 3221.00 | ECG: no myocardial ischaemia |
| 26973 | 3222.00 | ECG:shows myocardial ischaemia |
| 35287 | 322Z.00 | ECG: myocardial ischaemia NOS |
| 7783 | 323..00 | ECG: myocardial infarction |
| 26974 | 3231.00 | ECG: no myocardial infarction |
| 39904 | 3232.00 | ECG: old myocardial infarction |
| 59032 | 323Z.00 | ECG: myocardial infarct NOS |
| 39584 | 3889.00 | Euroscore for angina |
| 52637 | 388E.00 | Canadian Cardiovascular Society classification of angina |
| 57962 | 388F.00 | Cardiovascular Limitations and Symptoms Profile angina score |
| 107967 | 661M000 | Angina self-management plan agreed |
| 13185 | 662K.00 | Angina control |
| 19542 | 662K000 | Angina control - good |
| 15373 | 662K100 | Angina control - poor |
| 14782 | 662K200 | Angina control - improving |
| 29300 | 662K300 | Angina control - worsening |
| 15349 | 662Kz00 | Angina control NOS |
| 48980 | 66f0.00 | Cardiovascular disease annual review |
| 93400 | 679W.00 | Health education - cardiovascular disease |
| 18135 | 6A2..00 | Coronary heart disease annual review |
| 10260 | 6A4..00 | Coronary heart disease review |
| 61670 | 889A.00 | Diab mellit insulin-glucose infus acute myocardial infarct |
| 45960 | 8B27.00 | Antianginal therapy |
| 11648 | 8B3k.00 | Coronary heart disease medication review |
| 100496 | 8CEJ.00 | Coronary heart disease leaflet given |
| 103932 | 8CMP.00 | Coronary heart disease care plan |
| 95550 | 8H2V.00 | Admit ischaemic heart disease emergency |
| 94203 | 8I3z.00 | Cardiovascular disease annual review declined |
| 108056 | 8IEY.00 | Referral to Angina Plan self-management programme declined |
| 107574 | 8T04.00 | Referral to Angina Plan self-management programme |
| 100437 | 9hM..00 | Exception reporting: myocardial infarction quality indicator |
| 101695 | 9hM0.00 | Exc myocard infarction quality indicators: informed dissent |
| 24176 | A340000 | Streptococcal angina |
| 9607 | A740.00 | Herpangina |
| 240 | G3...00 | Ischaemic heart disease |
| 24783 | G3...11 | Arteriosclerotic heart disease |
| 20416 | G3...12 | Atherosclerotic heart disease |
| 1792 | G3...13 | IHD - Ischaemic heart disease |
| 241 | G30..00 | Acute myocardial infarction |
| 13566 | G30..11 | Attack - heart |
| 2491 | G30..12 | Coronary thrombosis |
| 30421 | G30..13 | Cardiac rupture following myocardial infarction (MI) |
| 1204 | G30..14 | Heart attack |
| 1677 | G30..15 | MI - acute myocardial infarction |
| 13571 | G30..16 | Thrombosis - coronary |
| 17689 | G30..17 | Silent myocardial infarction |
| 12139 | G300.00 | Acute anterolateral infarction |
| 5387 | G301.00 | Other specified anterior myocardial infarction |
| 17872 | G301100 | Acute anteroseptal infarction |
| 14897 | G301z00 | Anterior myocardial infarction NOS |
| 8935 | G302.00 | Acute inferolateral infarction |
| 29643 | G303.00 | Acute inferoposterior infarction |
| 23892 | G304.00 | Posterior myocardial infarction NOS |
| 14898 | G305.00 | Lateral myocardial infarction NOS |
| 63467 | G306.00 | True posterior myocardial infarction |
| 3704 | G307.00 | Acute subendocardial infarction |
| 9507 | G307000 | Acute non-Q wave infarction |
| 10562 | G307100 | Acute non-ST segment elevation myocardial infarction |
| 1678 | G308.00 | Inferior myocardial infarction NOS |
| 30330 | G309.00 | Acute Q-wave infarct |
| 17133 | G30A.00 | Mural thrombosis |
| 32854 | G30B.00 | Acute posterolateral myocardial infarction |
| 29758 | G30X.00 | Acute transmural myocardial infarction of unspecif site |
| 12229 | G30X000 | Acute ST segment elevation myocardial infarction |
| 34803 | G30y.00 | Other acute myocardial infarction |
| 28736 | G30y000 | Acute atrial infarction |
| 62626 | G30y100 | Acute papillary muscle infarction |
| 41221 | G30y200 | Acute septal infarction |
| 46017 | G30yz00 | Other acute myocardial infarction NOS |
| 14658 | G30z.00 | Acute myocardial infarction NOS |
| 27951 | G31..00 | Other acute and subacute ischaemic heart disease |
| 23579 | G310.00 | Postmyocardial infarction syndrome |
| 15661 | G310.11 | Dressler's syndrome |
| 36523 | G311.00 | Preinfarction syndrome |
| 4656 | G311.11 | Crescendo angina |
| 39655 | G311.12 | Impending infarction |
| 1431 | G311.13 | Unstable angina |
| 19655 | G311.14 | Angina at rest |
| 61072 | G311000 | Myocardial infarction aborted |
| 55137 | G311011 | MI - myocardial infarction aborted |
| 7347 | G311100 | Unstable angina |
| 17307 | G311200 | Angina at rest |
| 34328 | G311300 | Refractory angina |
| 18118 | G311400 | Worsening angina |
| 11983 | G311500 | Acute coronary syndrome |
| 54251 | G311z00 | Preinfarction syndrome NOS |
| 39449 | G312.00 | Coronary thrombosis not resulting in myocardial infarction |
| 9413 | G31y.00 | Other acute and subacute ischaemic heart disease |
| 9276 | G31y000 | Acute coronary insufficiency |
| 68357 | G31y100 | Microinfarction of heart |
| 39693 | G31y200 | Subendocardial ischaemia |
| 21844 | G31y300 | Transient myocardial ischaemia |
| 27977 | G31yz00 | Other acute and subacute ischaemic heart disease NOS |
| 4017 | G32..00 | Old myocardial infarction |
| 16408 | G32..11 | Healed myocardial infarction |
| 17464 | G32..12 | Personal history of myocardial infarction |
| 1430 | G33..00 | Angina pectoris |
| 20095 | G330.00 | Angina decubitus |
| 18125 | G330000 | Nocturnal angina |
| 29902 | G330z00 | Angina decubitus NOS |
| 12986 | G331.00 | Prinzmetal's angina |
| 11048 | G331.11 | Variant angina pectoris |
| 36854 | G332.00 | Coronary artery spasm |
| 25842 | G33z.00 | Angina pectoris NOS |
| 66388 | G33z000 | Status anginosus |
| 54535 | G33z100 | Stenocardia |
| 7696 | G33z200 | Syncope anginosa |
| 1414 | G33z300 | Angina on effort |
| 32450 | G33z400 | Ischaemic chest pain |
| 9555 | G33z500 | Post infarct angina |
| 26863 | G33z600 | New onset angina |
| 12804 | G33z700 | Stable angina |
| 28554 | G33zz00 | Angina pectoris NOS |
| 28138 | G34..00 | Other chronic ischaemic heart disease |
| 5413 | G340.00 | Coronary atherosclerosis |
| 1655 | G340.11 | Triple vessel disease of the heart |
| 1344 | G340.12 | Coronary artery disease |
| 3999 | G340000 | Single coronary vessel disease |
| 5254 | G340100 | Double coronary vessel disease |
| 36609 | G342.00 | Atherosclerotic cardiovascular disease |
| 7320 | G343.00 | Ischaemic cardiomyopathy |
| 29421 | G344.00 | Silent myocardial ischaemia |
| 34633 | G34y.00 | Other specified chronic ischaemic heart disease |
| 24540 | G34y000 | Chronic coronary insufficiency |
| 23078 | G34y100 | Chronic myocardial ischaemia |
| 35713 | G34yz00 | Other specified chronic ischaemic heart disease NOS |
| 15754 | G34z.00 | Other chronic ischaemic heart disease NOS |
| 18889 | G34z000 | Asymptomatic coronary heart disease |
| 18842 | G35..00 | Subsequent myocardial infarction |
| 45809 | G350.00 | Subsequent myocardial infarction of anterior wall |
| 38609 | G351.00 | Subsequent myocardial infarction of inferior wall |
| 72562 | G353.00 | Subsequent myocardial infarction of other sites |
| 46166 | G35X.00 | Subsequent myocardial infarction of unspecified site |
| 36423 | G36..00 | Certain current complication follow acute myocardial infarct |
| 24126 | G360.00 | Haemopericardium/current comp folow acut myocard infarct |
| 59189 | G363.00 | Ruptur cardiac wall w'out haemopericard/cur comp fol ac MI |
| 59940 | G364.00 | Ruptur chordae tendinae/curr comp fol acute myocard infarct |
| 69474 | G365.00 | Rupture papillary muscle/curr comp fol acute myocard infarct |
| 29553 | G366.00 | Thrombosis atrium,auric append&vent/curr comp foll acute MI |
| 32272 | G38..00 | Postoperative myocardial infarction |
| 46112 | G380.00 | Postoperative transmural myocardial infarction anterior wall |
| 46276 | G381.00 | Postoperative transmural myocardial infarction inferior wall |
| 106812 | G383.00 | Postoperative transmural myocardial infarction unspec site |
| 41835 | G384.00 | Postoperative subendocardial myocardial infarction |
| 68748 | G38z.00 | Postoperative myocardial infarction, unspecified |
| 22383 | G3y..00 | Other specified ischaemic heart disease |
| 1676 | G3z..00 | Ischaemic heart disease NOS |
| 22672 | G5yX.00 | Cardiovascular disease, unspecified |
| 52517 | Gyu3.00 | [X]Ischaemic heart diseases |
| 39546 | Gyu3000 | [X]Other forms of angina pectoris |
| 68401 | Gyu3200 | [X]Other forms of acute ischaemic heart disease |
| 47637 | Gyu3300 | [X]Other forms of chronic ischaemic heart disease |
| 96838 | Gyu3400 | [X]Acute transmural myocardial infarction of unspecif site |
| 109035 | Gyu3500 | [X]Subsequent myocardial infarction of other sites |
| 99991 | Gyu3600 | [X]Subsequent myocardial infarction of unspecified site |
| 99051 | Gyu5g00 | [X]Cardiovascular disease, unspecified |
| 17586 | J421.11 | Angina - abdominal |
| 58982 | L186.00 | Other cardiovascular diseases in pregnancy/childbirth/puerp |
| 55878 | Q494.00 | Transient myocardial ischaemia of newborn |
| 59350 | ZR37.00 | Canadian Cardiovascular Society classification of angina |
| 55673 | ZR3P.00 | CLASP angina score |
| 57910 | ZR3P.11 | CLASP angina score |
| 98295 | ZRB1.00 | Euroscore for angina |
| 51043 | ZRBN.00 | Duke's coronary artery disease score |
| 24512 | ZV71700 | [V]Observation for suspected cardiovascular disease |
| 12541 | ZV71900 | [V]Observation for suspected myocardial infarction |
| Gout codes | |  |
| 16475 | 669..00 | Gout monitoring |
| 52117 | 669Z.00 | Gout monitoring NOS |
| 52103 | 6698.00 | Gout drug side effects |
| 11281 | ZV77500 | [V]Screening for gout |
| 16145 | 6876.00 | Gout screen |
| 61145 | C341z00 | Gouty nephropathy NOS |
| 24153 | C34z.00 | Gout NOS |
| 97539 | N023200 | Gouty arthritis of the upper arm |
| 93689 | C34y100 | Gouty tophi of heart |
| 4440 | C34y200 | Gouty tophi of other sites |
| 68209 | 6696.00 | Date of last gout attack |
| 29658 | 6693.00 | Joints gout affected |
| 49775 | N023600 | Gouty arthritis of the lower leg |
| 12594 | N023z00 | Gouty arthritis NOS |
| 2857 | N023.00 | Gouty arthritis |
| 34105 | 6699.00 | Gout treatment changed |
| 45465 | N023300 | Gouty arthritis of the forearm |
| 60541 | N023y00 | Gouty arthritis of other specified site |
| 58064 | N023x00 | Gouty arthritis of multiple sites |
| 94539 | Nyu1700 | [X]Other secondary gout |
| 59344 | C34y400 | Gouty neuritis |
| 57334 | G557300 | Gouty tophi of heart |
| 21687 | C345.00 | Gout due to impairment of renal function |
| 9874 | C34y500 | Gouty tophi of hand |
| 44566 | C344.00 | Drug-induced gout |
| 93677 | N023800 | Gouty arthritis of toe |
| 72471 | N023100 | Gouty arthritis of the shoulder region |
| 34006 | 6695.00 | Date gout treatment started |
| 52101 | N023400 | Gouty arthritis of the hand |
| 27521 | C34yz00 | Other specified gouty manifestation NOS |
| 10080 | C340.00 | Gouty arthropathy |
| 36481 | C34y000 | Gouty tophi of ear |
| 58746 | 6697.00 | Gout associated problems |
| 43646 | 669A.00 | Date gout treatment stopped |
| 14996 | 6691.00 | Initial gout assessment |
| 50067 | C34y300 | Gouty iritis |
| 709 | C34..00 | Gout |
| 35660 | 6692.00 | Follow-up gout assessment |
| 11462 | C342.00 | Idiopathic gout |
| 35664 | N023700 | Gouty arthritis of the ankle and foot |
| 3759 | 1443.00 | H/O: gout |
| 52969 | C341.00 | Gouty nephropathy |
| 28999 | C34y.00 | Other specified gouty manifestation |
| Type II diabetes codes | | |
| 506 | C100112 | Non-insulin dependent diabetes mellitus |
| 608 | 66A2.00 | Follow-up diabetic assessment |
| 711 | C10..00 | Diabetes mellitus |
| 758 | C10F.00 | Type 2 diabetes mellitus |
| 1323 | F420.00 | Diabetic retinopathy |
| 1407 | C10FJ00 | Insulin treated Type 2 diabetes mellitus |
| 1682 | C101.00 | Diabetes mellitus with ketoacidosis |
| 1684 | 66A4.00 | Diabetic on oral treatment |
| 2340 | F381311 | Diabetic amyotrophy |
| 2342 | F372.12 | Diabetic neuropathy |
| 2378 | 66AJ.00 | Diabetic - poor control |
| 2379 | 9N1Q.00 | Seen in diabetic clinic |
| 2471 | K01x100 | Nephrotic syndrome in diabetes mellitus |
| 2475 | C104.11 | Diabetic nephropathy |
| 2664 | L180900 | Gestational diabetes mellitus |
| 2986 | F420200 | Preproliferative diabetic retinopathy |
| 3286 | F420100 | Proliferative diabetic retinopathy |
| 3550 | 66A..00 | Diabetic monitoring |
| 3837 | F420400 | Diabetic maculopathy |
| 4513 | C109.00 | Non-insulin dependent diabetes mellitus |
| 5002 | F372.11 | Diabetic polyneuropathy |
| 5884 | C109.11 | NIDDM - Non-insulin dependent diabetes mellitus |
| 6125 | 66AS.00 | Diabetic annual review |
| 6430 | 9NM0.00 | Attending diabetes clinic |
| 6813 | 1434.00 | H/O: diabetes mellitus |
| 7045 | 14F4.00 | H/O: Admission in last year for diabetes foot problem |
| 7059 | 8H2J.00 | Admit diabetic emergency |
| 7069 | F420000 | Background diabetic retinopathy |
| 7328 | M037200 | Cellulitis in diabetic foot |
| 7563 | 66A3.00 | Diabetic on diet only |
| 7795 | C106.12 | Diabetes mellitus with neuropathy |
| 8306 | 8H7f.00 | Referral to diabetes nurse |
| 8403 | C109700 | Non-insulin dependent diabetes mellitus - poor control |
| 8414 | 8CA4100 | Pt advised re diabetic diet |
| 8446 | L180811 | Gestational diabetes mellitus |
| 8618 | ZLA2500 | Seen by diabetic liaison nurse |
| 8836 | 66AR.00 | Diabetes management plan given |
| 8842 | 66A5.00 | Diabetic on insulin |
| 9013 | 66AJ.11 | Unstable diabetes |
| 9145 | 9N4I.00 | DNA - Did not attend diabetic clinic |
| 9835 | 2BBL.00 | O/E - diabetic maculopathy present both eyes |
| 9881 | M271200 | Mixed diabetic ulcer - foot |
| 9897 | 9OL..00 | Diabetes monitoring admin. |
| 9958 | 42W..00 | Hb. A1C - diabetic control |
| 9974 | 9N1v.00 | Seen in diabetic eye clinic |
| 10098 | C10yy00 | Other specified diabetes mellitus with other spec comps |
| 10099 | F420300 | Advanced diabetic maculopathy |
| 10278 | L180800 | Diabetes mellitus arising in pregnancy |
| 10642 | ZC2C800 | Dietary advice for diabetes mellitus |
| 10659 | F464000 | Diabetic cataract |
| 10755 | F420600 | Non proliferative diabetic retinopathy |
| 10824 | 9N1i.00 | Seen in diabetic foot clinic |
| 11018 | 8HBG.00 | Diabetic retinopathy 12 month review |
| 11041 | 9h41.00 | Excepted from diabetes qual indicators: Patient unsuitable |
| 11129 | 2BBQ.00 | O/E - left eye background diabetic retinopathy |
| 11149 | R102.11 | [D]Prediabetes |
| 11348 | 9h42.00 | Excepted from diabetes quality indicators: Informed dissent |
| 11359 | L180.00 | Diabetes mellitus during pregnancy/childbirth/puerperium |
| 11433 | 2BBP.00 | O/E - right eye background diabetic retinopathy |
| 11471 | 8B3l.00 | Diabetes medication review |
| 11551 | C10B.00 | Diabetes mellitus induced by steroids |
| 11599 | 7276.00 | Pan retinal photocoagulation for diabetes |
| 11626 | F420z00 | Diabetic retinopathy NOS |
| 11663 | M271100 | Neuropathic diabetic ulcer - foot |
| 11848 | C314.11 | Renal diabetes |
| 11930 | 9NN9.00 | Under care of diabetes specialist nurse |
| 11977 | ZL62500 | Referral to diabetes nurse |
| 12030 | 9OL6.00 | Diabetes monitoring 3rd letter |
| 12213 | 8BL2.00 | Patient on maximal tolerated therapy for diabetes |
| 12225 | 8H7C.00 | Refer, diabetic liaison nurse |
| 12247 | 8I6G.00 | Diabetic foot examination not indicated |
| 12262 | 8I3X.00 | Diabetic retinopathy screening refused |
| 12307 | 66AU.00 | Diabetes care by hospital only |
| 12506 | 66AP.00 | Diabetes: practice programme |
| 12507 | 9N2i.00 | Seen by diabetic liaison nurse |
| 12640 | C10FC00 | Type 2 diabetes mellitus with nephropathy |
| 12675 | 66AQ.00 | Diabetes: shared care programme |
| 12682 | 679R.00 | Patient offered diabetes structured education programme |
| 12703 | 3881.00 | Education score - diabetes |
| 12736 | C10F500 | Type 2 diabetes mellitus with gangrene |
| 13057 | 679L.00 | Health education - diabetes |
| 13067 | 66AZ.00 | Diabetic monitoring NOS |
| 13069 | 66A8.00 | Has seen dietician - diabetes |
| 13070 | 66A1.00 | Initial diabetic assessment |
| 13071 | 66AI.00 | Diabetic - good control |
| 13074 | 13B1.00 | Diabetic diet |
| 13078 | 13AC.00 | Diabetic weight reducing diet |
| 13097 | 2BBT.00 | O/E - right eye proliferative diabetic retinopathy |
| 13099 | 2BBR.00 | O/E - right eye preproliferative diabetic retinopathy |
| 13100 | 2BBJ.00 | O/E - no right diabetic retinopathy |
| 13101 | 2BBV.00 | O/E - left eye proliferative diabetic retinopathy |
| 13102 | 2BBW.00 | O/E - right eye diabetic maculopathy |
| 13103 | 2BBS.00 | O/E - left eye preproliferative diabetic retinopathy |
| 13104 | 2BBK.00 | O/E - no left diabetic retinopathy |
| 13108 | 2BBX.00 | O/E - left eye diabetic maculopathy |
| 13191 | 9OL..11 | Diabetes clinic administration |
| 13192 | 9OLA.00 | Diabetes monitor. check done |
| 13194 | 9OL4.00 | Diabetes monitoring 1st letter |
| 13195 | 9OL5.00 | Diabetes monitoring 2nd letter |
| 13196 | 66AD.00 | Fundoscopy - diabetic check |
| 13197 | 9OL1.00 | Attends diabetes monitoring |
| 13279 | C104y00 | Other specified diabetes mellitus with renal complications |
| 13678 | ZL62600 | Referral to diabetic liaison nurse |
| 14049 | 42WZ.00 | Hb. A1C - diabetic control NOS |
| 14050 | 42c..00 | HbA1 - diabetic control |
| 14803 | C100100 | Diabetes mellitus, adult onset, no mention of complication |
| 14889 | C100111 | Maturity onset diabetes |
| 15690 | C103.00 | Diabetes mellitus with ketoacidotic coma |
| 16230 | C106.00 | Diabetes mellitus with neurological manifestation |
| 16490 | 66AH.00 | Diabetic treatment changed |
| 16491 | C106.13 | Diabetes mellitus with polyneuropathy |
| 16502 | C104.00 | Diabetes mellitus with renal manifestation |
| 16881 | ZV65312 | [V]Dietary counselling in diabetes mellitus |
| 16946 | 13L4.11 | Diabetic child |
| 17067 | F171100 | Autonomic neuropathy due to diabetes |
| 17095 | 2G5A.00 | O/E - Right diabetic foot at risk |
| 17247 | F35z000 | Diabetic mononeuritis NOS |
| 17262 | C109600 | Non-insulin-dependent diabetes mellitus with retinopathy |
| 17313 | F440700 | Diabetic iritis |
| 17859 | C109.12 | Type 2 diabetes mellitus |
| 17869 | 66AL.00 | Diabetic-uncooperative patient |
| 17886 | 66AM.00 | Diabetic - follow-up default |
| 18056 | 2G5C.00 | Foot abnormality - diabetes related |
| 18066 | 8CE0.00 | Diabetic leaflet given |
| 18142 | N030000 | Diabetic cheiroarthropathy |
| 18143 | C109G11 | Type II diabetes mellitus with arthropathy |
| 18167 | 66AT.00 | Annual diabetic blood test |
| 18185 | 2G5D.00 | Foot abnormality - non-diabetes |
| 18209 | C109012 | Type 2 diabetes mellitus with renal complications |
| 18219 | C109.13 | Type II diabetes mellitus |
| 18264 | C109J12 | Insulin treated Type II diabetes mellitus |
| 18278 | C109J00 | Insulin treated Type 2 diabetes mellitus |
| 18311 | 68A7.00 | Diabetic retinopathy screening |
| 18390 | C10FM00 | Type 2 diabetes mellitus with persistent microalbuminuria |
| 18425 | C10FB00 | Type 2 diabetes mellitus with polyneuropathy |
| 18496 | C10F600 | Type 2 diabetes mellitus with retinopathy |
| 18662 | 8HBH.00 | Diabetic retinopathy 6 month review |
| 18747 | 8I6F.00 | Diabetic retinopathy screening not indicated |
| 18777 | C10F000 | Type 2 diabetes mellitus with renal complications |
| 18824 | 8I3W.00 | Diabetic foot examination declined |
| 19381 | 8HTk.00 | Referral to diabetic eye clinic |
| 19739 | 68A9.00 | Diabetic retinopathy screening offered |
| 20696 | 66AA.11 | Injection sites - diabetic |
| 20900 | 9OLA.11 | Diabetes monitored |
| 21472 | Q441.00 | Neonatal diabetes mellitus |
| 21482 | C102.00 | Diabetes mellitus with hyperosmolar coma |
| 21689 | 13AB.00 | Diabetic lipid lowering diet |
| 22023 | 66AJz00 | Diabetic - poor control NOS |
| 22130 | 9OL3.00 | Diabetes monitoring default |
| 22487 | C10N.00 | Secondary diabetes mellitus |
| 22573 | C106z00 | Diabetes mellitus NOS with neurological manifestation |
| 22823 | 66Ab.00 | Diabetic foot examination |
| 22884 | C10F.11 | Type II diabetes mellitus |
| 22967 | 2BBF.00 | Retinal abnormality - diabetes related |
| 23479 | C350011 | Bronzed diabetes |
| 24327 | M271000 | Ischaemic ulcer diabetic foot |
| 24363 | 8A13.00 | Diabetic stabilisation |
| 24458 | C109711 | Type II diabetes mellitus - poor control |
| 24490 | C100000 | Diabetes mellitus, juvenile type, no mention of complication |
| 24571 | F372200 | Asymptomatic diabetic neuropathy |
| 24693 | C109G00 | Non-insulin dependent diabetes mellitus with arthropathy |
| 24836 | C109C12 | Type 2 diabetes mellitus with nephropathy |
| 25041 | ZC2CA00 | Dietary advice for type II diabetes |
| 25591 | C10FQ00 | Type 2 diabetes mellitus with exudative maculopathy |
| 25627 | C10F700 | Type 2 diabetes mellitus - poor control |
| 25636 | 66Aa.00 | Diabetic diet - poor compliance |
| 26054 | C10FL00 | Type 2 diabetes mellitus with persistent proteinuria |
| 26108 | C10B000 | Steroid induced diabetes mellitus without complication |
| 26603 | 9OL2.00 | Refuses diabetes monitoring |
| 26604 | 66AY.00 | Diabetic diet - good compliance |
| 26605 | 9OLB.00 | Attended diabetes structured education programme |
| 26664 | 2G5B.00 | O/E - Left diabetic foot at risk |
| 26665 | 2G51100 | Foot abnormality - non-diabetes |
| 26666 | 2G5E.00 | O/E - Right diabetic foot at low risk |
| 26667 | 2G5I.00 | O/E - Left diabetic foot at low risk |
| 27891 | N030100 | Diabetic Charcot arthropathy |
| 27921 | 2G51000 | Foot abnormality - diabetes related |
| 28574 | 9h4..00 | Exception reporting: diabetes quality indicators |
| 28769 | 66AV.00 | Diabetic on insulin and oral treatment |
| 28856 | 8CP2.00 | Transition of diabetes care options discussed |
| 28873 | 66Ai.00 | Diabetic 6 month review |
| 29041 | 66AN.00 | Date diabetic treatment start |
| 29979 | C109900 | Non-insulin-dependent diabetes mellitus without complication |
| 30477 | F420700 | High risk proliferative diabetic retinopathy |
| 30648 | 9N4p.00 | Did not attend diabetic retinopathy clinic |
| 30970 | Q44B.00 | Syndrome of infant of mother with gestational diabetes |
| 31053 | R054300 | [D]Widespread diabetic foot gangrene |
| 31141 | 9OL8.00 | Diabetes monitor.phone invite |
| 31156 | 2G5J.00 | O/E - Left diabetic foot at moderate risk |
| 31157 | 2G5F.00 | O/E - Right diabetic foot at moderate risk |
| 31171 | 2G5G.00 | O/E - Right diabetic foot at high risk |
| 31172 | 2G5K.00 | O/E - Left diabetic foot at high risk |
| 31240 | 9OL7.00 | Diabetes monitor.verbal invite |
| 31241 | 9OLZ.00 | Diabetes monitoring admin.NOS |
| 31790 | F372.00 | Polyneuropathy in diabetes |
| 32193 | C11y000 | Steroid induced diabetes |
| 32403 | C107.11 | Diabetes mellitus with gangrene |
| 32556 | C107.12 | Diabetes with gangrene |
| 32619 | 66Af.00 | Patient diabetes education review |
| 32627 | C10FN00 | Type 2 diabetes mellitus with ketoacidosis |
| 32739 | 9N0n.00 | Seen in community diabetes specialist clinic |
| 32999 | Q440.00 | 'Infant of a diabetic mother' syndrome |
| 33254 | C105.00 | Diabetes mellitus with ophthalmic manifestation |
| 33343 | C10y.00 | Diabetes mellitus with other specified manifestation |
| 33807 | C107200 | Diabetes mellitus, adult with gangrene |
| 33969 | C10A100 | Malnutrition-related diabetes mellitus with ketoacidosis |
| 34152 | G73y000 | Diabetic peripheral angiopathy |
| 34268 | C10F200 | Type 2 diabetes mellitus with neurological complications |
| 34283 | C105z00 | Diabetes mellitus NOS with ophthalmic manifestation |
| 34450 | C10FK00 | Hyperosmolar non-ketotic state in type 2 diabetes mellitus |
| 34528 | 3882.00 | Diabetes well being questionnaire |
| 34639 | L180100 | Diabetes mellitus during pregnancy - baby delivered |
| 34912 | C109400 | Non-insulin dependent diabetes mellitus with ulcer |
| 35105 | C104100 | Diabetes mellitus, adult onset, with renal manifestation |
| 35107 | C104z00 | Diabetes mellitus with nephropathy NOS |
| 35116 | 2G5L.00 | O/E - Left diabetic foot - ulcerated |
| 35316 | 2G5H.00 | O/E - Right diabetic foot - ulcerated |
| 35321 | 8H3O.00 | Non-urgent diabetic admission |
| 35383 | 9OLD.00 | Diabetic patient unsuitable for digital retinal photography |
| 35385 | C10FH00 | Type 2 diabetes mellitus with neuropathic arthropathy |
| 35399 | C107.00 | Diabetes mellitus with peripheral circulatory disorder |
| 35785 | F372100 | Chronic painful diabetic neuropathy |
| 36633 | C109K00 | Hyperosmolar non-ketotic state in type 2 diabetes mellitus |
| 36669 | 66b1.00 | Diabetic monitoring not required |
| 36695 | C10D.00 | Diabetes mellitus autosomal dominant type 2 |
| 36855 | 2BBG.00 | Retinal abnormality - non-diabetes |
| 37315 | F3y0.00 | Diabetic mononeuropathy |
| 37648 | C109J11 | Insulin treated non-insulin dependent diabetes mellitus |
| 37806 | C10FF00 | Type 2 diabetes mellitus with peripheral angiopathy |
| 38078 | 66A9.00 | Understands diet - diabetes |
| 38103 | 9N0m.00 | Seen in diabetic nurse consultant clinic |
| 38129 | 9N0o.00 | Seen in community diabetic specialist nurse clinic |
| 38130 | ZRB6.00 | Diabetes wellbeing questionnaire |
| 38617 | C101y00 | Other specified diabetes mellitus with ketoacidosis |
| 38986 | C100.00 | Diabetes mellitus with no mention of complication |
| 39317 | C106100 | Diabetes mellitus, adult onset, + neurological manifestation |
| 39420 | F381300 | Myasthenic syndrome due to diabetic amyotrophy |
| 40023 | C102000 | Diabetes mellitus, juvenile type, with hyperosmolar coma |
| 40401 | C109500 | Non-insulin dependent diabetes mellitus with gangrene |
| 41389 | C105100 | Diabetes mellitus, adult onset, + ophthalmic manifestation |
| 41686 | Cyu2000 | [X]Other specified diabetes mellitus |
| 42505 | C101z00 | Diabetes mellitus NOS with ketoacidosis |
| 42567 | C103000 | Diabetes mellitus, juvenile type, with ketoacidotic coma |
| 42762 | C109612 | Type 2 diabetes mellitus with retinopathy |
| 43139 | C102100 | Diabetes mellitus, adult onset, with hyperosmolar coma |
| 43227 | C10F311 | Type II diabetes mellitus with multiple complications |
| 43453 | C10C.00 | Diabetes mellitus autosomal dominant |
| 43785 | C109D00 | Non-insulin dependent diabetes mellitus with hypoglyca coma |
| 43857 | C10M.00 | Lipoatrophic diabetes mellitus |
| 43951 | 66AK.00 | Diabetic - cooperative patient |
| 44033 | F345000 | Diabetic mononeuritis multiplex |
| 44312 | 9M10.00 | Informed dissent for diabetes national audit |
| 44779 | C109E12 | Type 2 diabetes mellitus with diabetic cataract |
| 44982 | C10FE00 | Type 2 diabetes mellitus with diabetic cataract |
| 45250 | ZL22500 | Under care of diabetic liaison nurse |
| 45467 | C109B00 | Non-insulin dependent diabetes mellitus with polyneuropathy |
| 45491 | C10z.00 | Diabetes mellitus with unspecified complication |
| 45913 | C109712 | Type 2 diabetes mellitus - poor control |
| 45919 | C109212 | Type 2 diabetes mellitus with neurological complications |
| 46150 | C109512 | Type 2 diabetes mellitus with gangrene |
| 46290 | C108y00 | Other specified diabetes mellitus with multiple comps |
| 46533 | 13Y1.00 | Diabetic association member |
| 46577 | 66AX.00 | Diabetes: shared care in pregnancy - diabetol and obstet |
| 46624 | C10C.11 | Maturity onset diabetes in youth |
| 46917 | C10FD00 | Type 2 diabetes mellitus with hypoglycaemic coma |
| 47011 | 8Hj0.00 | Referral to diabetes structured education programme |
| 47032 | 8CS0.00 | Diabetes care plan agreed |
| 47058 | 8Hg4.00 | Discharged from care of diabetes specialist nurse |
| 47144 | 2BBM.00 | O/E - diabetic maculopathy absent both eyes |
| 47315 | C10F711 | Type II diabetes mellitus - poor control |
| 47321 | C10F100 | Type 2 diabetes mellitus with ophthalmic complications |
| 47328 | 2BBk.00 | O/E - right eye stable treated prolif diabetic retinopathy |
| 47341 | 8A12.00 | Diabetic crisis monitoring |
| 47377 | C105y00 | Other specified diabetes mellitus with ophthalmic complicatn |
| 47409 | C109B11 | Type II diabetes mellitus with polyneuropathy |
| 47584 | F420500 | Advanced diabetic retinal disease |
| 47816 | C109H11 | Type II diabetes mellitus with neuropathic arthropathy |
| 47954 | C10F900 | Type 2 diabetes mellitus without complication |
| 48078 | F372000 | Acute painful diabetic neuropathy |
| 48192 | C109E11 | Type II diabetes mellitus with diabetic cataract |
| 49074 | C10F400 | Type 2 diabetes mellitus with ulcer |
| 49559 | L180300 | Diabetes mellitus during pregnancy - baby not yet delivered |
| 49640 | 2G5W.00 | O/E - left chronic diabetic foot ulcer |
| 49655 | C10F611 | Type II diabetes mellitus with retinopathy |
| 49869 | C109G12 | Type 2 diabetes mellitus with arthropathy |
| 49884 | 6761.00 | Diabetic pre-pregnancy counselling |
| 50064 | Q44y100 | Transitory metabolic disturbance-infant pre-diabetic mother |
| 50175 | 66AW.00 | Diabetic foot risk assessment |
| 50225 | C109011 | Type II diabetes mellitus with renal complications |
| 50429 | C109100 | Non-insulin-dependent diabetes mellitus with ophthalm comps |
| 50527 | C10FB11 | Type II diabetes mellitus with polyneuropathy |
| 50609 | L180600 | Pre-existing diabetes mellitus, non-insulin-dependent |
| 50813 | C109A11 | Type II diabetes mellitus with mononeuropathy |
| 50937 | 8HTe.00 | Referral to diabetes preconception counselling clinic |
| 50960 | L180500 | Pre-existing diabetes mellitus, insulin-dependent |
| 50972 | C100z00 | Diabetes mellitus NOS with no mention of complication |
| 51066 | 9OLC.00 | Family/carer attended diabetes structured education prog |
| 51697 | C10G.00 | Secondary pancreatic diabetes mellitus |
| 51756 | C10FP00 | Type 2 diabetes mellitus with ketoacidotic coma |
| 52041 | 2BBl.00 | O/E - left eye stable treated prolif diabetic retinopathy |
| 52212 | Cyu2.00 | [X]Diabetes mellitus |
| 52236 | C10A.00 | Malnutrition-related diabetes mellitus |
| 52237 | 9360.00 | Patient held diabetic record issued |
| 52303 | C109000 | Non-insulin-dependent diabetes mellitus with renal comps |
| 52630 | 2BBo.00 | O/E - sight threatening diabetic retinopathy |
| 53200 | C101000 | Diabetes mellitus, juvenile type, with ketoacidosis |
| 53238 | 66AG.00 | Diabetic drug side effects |
| 53392 | C10F911 | Type II diabetes mellitus without complication |
| 53634 | R054200 | [D]Gangrene of toe in diabetic |
| 54419 | 918T.00 | Diabetes key contact |
| 54846 | 9OL9.00 | Diabetes monitoring deleted |
| 54856 | C101100 | Diabetes mellitus, adult onset, with ketoacidosis |
| 54899 | C109F11 | Type II diabetes mellitus with peripheral angiopathy |
| 55075 | C109411 | Type II diabetes mellitus with ulcer |
| 55123 | 66AO.00 | Date diabetic treatment stopp. |
| 55431 | L180X00 | Pre-existing diabetes mellitus, unspecified |
| 55842 | C109200 | Non-insulin-dependent diabetes mellitus with neuro comps |
| 56268 | C109D11 | Type II diabetes mellitus with hypoglycaemic coma |
| 57278 | C10F011 | Type II diabetes mellitus with renal complications |
| 57333 | N030011 | Diabetic cheiropathy |
| 57389 | 93C4.00 | Patient consent given for addition to diabetic register |
| 57723 | 8HHy.00 | Referral to diabetic register |
| 58133 | ZLD7500 | Discharge by diabetic liaison nurse |
| 58604 | C109611 | Type II diabetes mellitus with retinopathy |
| 58639 | 8I57.00 | Patient held diabetic record declined |
| 59253 | C10FG00 | Type 2 diabetes mellitus with arthropathy |
| 59288 | C103y00 | Other specified diabetes mellitus with coma |
| 59365 | C109C00 | Non-insulin dependent diabetes mellitus with nephropathy |
| 59725 | C109111 | Type II diabetes mellitus with ophthalmic complications |
| 59903 | C106.11 | Diabetic amyotrophy |
| 59991 | C10D.11 | Maturity onset diabetes in youth type 2 |
| 60699 | C109F12 | Type 2 diabetes mellitus with peripheral angiopathy |
| 60796 | C10FL11 | Type II diabetes mellitus with persistent proteinuria |
| 61071 | C109D12 | Type 2 diabetes mellitus with hypoglycaemic coma |
| 61122 | C10H.00 | Diabetes mellitus induced by non-steroid drugs |
| 61210 | TJ23z00 | Adverse reaction to insulins and antidiabetic agents NOS |
| 61461 | 9M00.00 | Informed consent for diabetes national audit |
| 61470 | 66Al.00 | Diabetic monitoring - higher risk albumin excretion |
| 61523 | C106y00 | Other specified diabetes mellitus with neurological comps |
| 62107 | C109511 | Type II diabetes mellitus with gangrene |
| 62146 | C109300 | Non-insulin-dependent diabetes mellitus with multiple comps |
| 62384 | 2G5V.00 | O/E - right chronic diabetic foot ulcer |
| 62674 | C10FA00 | Type 2 diabetes mellitus with mononeuropathy |
| 63357 | C107100 | Diabetes mellitus, adult, + peripheral circulatory disorder |
| 63371 | C10y100 | Diabetes mellitus, adult, + other specified manifestation |
| 63412 | 8CR2.00 | Diabetes clinical management plan |
| 63690 | C10FR00 | Type 2 diabetes mellitus with gastroparesis |
| 63762 | C10z100 | Diabetes mellitus, adult onset, + unspecified complication |
| 64142 | 8Hl1.00 | Referral for diabetic retinopathy screening |
| 64283 | C10zy00 | Other specified diabetes mellitus with unspecified comps |
| 64357 | C10zz00 | Diabetes mellitus NOS with unspecified complication |
| 64384 | L180z00 | Diabetes mellitus in pregnancy/childbirth/puerperium NOS |
| 64449 | C108z00 | Unspecified diabetes mellitus with multiple complications |
| 64571 | C109C11 | Type II diabetes mellitus with nephropathy |
| 64668 | C10FJ11 | Insulin treated Type II diabetes mellitus |
| 65025 | C107z00 | Diabetes mellitus NOS with peripheral circulatory disorder |
| 65062 | C103z00 | Diabetes mellitus NOS with ketoacidotic coma |
| 65267 | C10F300 | Type 2 diabetes mellitus with multiple complications |
| 65463 | F420800 | High risk non proliferative diabetic retinopathy |
| 65684 | U602311 | [X] Adverse reaction to insulins and antidiabetic agents |
| 65704 | C109412 | Type 2 diabetes mellitus with ulcer |
| 66475 | 66Ak.00 | Diabetic monitoring - lower risk albumin excretion |
| 66675 | C10A000 | Malnutrition-related diabetes mellitus with coma |
| 66965 | C109H12 | Type 2 diabetes mellitus with neuropathic arthropathy |
| 67635 | L180000 | Diabetes mellitus - unspec whether in pregnancy/puerperium |
| 67664 | ZRBa.00 | Education score - diabetes |
| 67853 | C106000 | Diabetes mellitus, juvenile, + neurological manifestation |
| 67905 | C109211 | Type II diabetes mellitus with neurological complications |
| 68546 | ZRB4.00 | Diabetes clinic satisfaction questionnaire |
| 68714 | SL23.00 | Insulins and antidiabetic poisoning |
| 68792 | C10z000 | Diabetes mellitus, juvenile type, + unspecified complication |
| 68818 | ZRB5.11 | DTSQ - Diabetes treatment satisfaction questionnaire |
| 68843 | C103100 | Diabetes mellitus, adult onset, with ketoacidotic coma |
| 68928 | TJ23.00 | Adverse reaction to insulins and antidiabetic agents |
| 69163 | 8HTi.00 | Referral to multidisciplinary diabetic clinic |
| 69278 | C109E00 | Non-insulin depend diabetes mellitus with diabetic cataract |
| 69748 | C105000 | Diabetes mellitus, juvenile type, + ophthalmic manifestation |
| 70316 | C109112 | Type 2 diabetes mellitus with ophthalmic complications |
| 70448 | C107000 | Diabetes mellitus, juvenile +peripheral circulatory disorder |
| 70821 | C10yz00 | Diabetes mellitus NOS with other specified manifestation |
| 72320 | C109A00 | Non-insulin dependent diabetes mellitus with mononeuropathy |
| 72345 | C102z00 | Diabetes mellitus NOS with hyperosmolar coma |
| 82474 | 8Hl4.00 | Referral to community diabetes specialist nurse |
| 83532 | 66Ao.00 | Diabetes type 2 review |
| 85660 | 66An.00 | Diabetes type 1 review |
| 85991 | C10FM11 | Type II diabetes mellitus with persistent microalbuminuria |
| 91164 | ZRB4.11 | CSQ - Diabetes clinic satisfaction questionnaire |
| 91646 | C10F411 | Type II diabetes mellitus with ulcer |
| 93380 | C10N100 | Cystic fibrosis related diabetes mellitus |
| 93390 | 9OLH.00 | Attended DAFNE diabetes structured education programme |
| 93491 | 9OLJ.00 | DAFNE diabetes structured education programme completed |
| 93529 | 9OLK.00 | DESMOND diabetes structured education programme completed |
| 93631 | 9OLL.00 | XPERT diabetes structured education programme completed |
| 93657 | 8Hj4.00 | Referral to DESMOND diabetes structured education programme |
| 93704 | 8Hj3.00 | Referral to DAFNE diabetes structured education programme |
| 93727 | C10FE11 | Type II diabetes mellitus with diabetic cataract |
| 93854 | 9OLM.00 | Diabetes structured education programme declined |
| 93870 | 8Hj5.00 | Referral to XPERT diabetes structured education programme |
| 93922 | C104000 | Diabetes mellitus, juvenile type, with renal manifestation |
| 94011 | 9OLG.00 | Attended XPERT diabetes structured education programme |
| 94186 | 9OLF.00 | Diabetes structured education programme completed |
| 94330 | 8H4e.00 | Referral to diabetes special interest general practitioner |
| 94383 | C10N000 | Secondary diabetes mellitus without complication |
| 94699 | ZRB5.00 | Diabetes treatment satisfaction questionnaire |
| 94777 | ZV13F00 | [V]Personal history of gestational diabetes mellitus |
| 94955 | 9NiE.00 | Did not attend XPERT diabetes structured education programme |
| 94956 | 8I84.00 | Did not complete XPERT diabetes structured education program |
| 95093 | 8I83.00 | Did not complete DESMOND diabetes structured educat program |
| 95094 | 8I81.00 | Did not complete diabetes structured education programme |
| 95159 | 9NiD.00 | Did not attend DESMOND diabetes structured education program |
| 95351 | C10FA11 | Type II diabetes mellitus with mononeuropathy |
| 95539 | C10FS00 | Maternally inherited diabetes mellitus |
| 95553 | 9NiA.00 | Did not attend diabetes structured education programme |
| 95636 | C10ER00 | Latent autoimmune diabetes mellitus in adult |
| 95641 | 8Hj1.00 | Family/carer referral to diabetes structured education prog |
| 95813 | 9N1o.00 | Seen in multidisciplinary diabetic clinic |
| 96506 | C10G000 | Secondary pancreatic diabetes mellitus without complication |
| 96823 | L180400 | Diabetes mellitus in pueperium - baby previously delivered |
| 97281 | 9Nl4.00 | Seen by general practitioner special interest in diabetes |
| 97809 | 8I82.00 | Did not complete DAFNE diabetes structured education program |
| 97824 | ZRB6.11 | DWBQ - Diabetes wellbeing questionnaire |
| 98392 | C10C.12 | Maturity onset diabetes in youth type 1 |
| 98616 | C10F211 | Type II diabetes mellitus with neurological complications |
| 98723 | C10FD11 | Type II diabetes mellitus with hypoglycaemic coma |
| 98954 | 3883.00 | Diabetes treatment satisfaction questionnaire |
| 99277 | 9NiC.00 | Did not attend DAFNE diabetes structured education programme |
| 99628 | Kyu0300 | [X]Glomerular disorders in diabetes mellitus |
| 99822 | 38DK.00 | Finnish diabetes risk score |
| 100033 | U60231E | [X] Adverse reaction to insulins and antidiabetic agents NOS |
| 100292 | Cyu2300 | [X]Unspecified diabetes mellitus with renal complications |
| 100347 | C10A500 | Malnutritn-relat diabetes melitus wth periph circul complctn |
| 100422 | 8HgC.00 | Discharged from diabetes shared care programme |
| 100436 | 679L000 | Education in self management of diabetes |
| 100533 | 66AQ000 | Unsuitable for diabetes year of care programme |
| 100964 | C10F111 | Type II diabetes mellitus with ophthalmic complications |
| 101177 | 66At.00 | Diabetic dietary review |
| 101190 | 66AQ100 | Declined consent for diabetes year of care programme |
| 101455 | 9OLN.00 | Diabetes monitor invitation by SMS (short message service) |
| 101456 | 8IAs.00 | Diabetic dietary review declined |
| 101728 | 66As.00 | Diabetic on subcutaneous treatment |
| 101801 | 66At100 | Type II diabetic dietary review |
| 101834 | 9h43.00 | Excepted from diabetes qual indicators: service unavailable |
| 101881 | 2BBr.00 | Impaired vision due to diabetic retinopathy |
| 102201 | C10FC11 | Type II diabetes mellitus with nephropathy |
| 102434 | 66Au.00 | Diabetic erectile dysfunction review |
| 102435 | 8CE0000 | Gestational diabetes information leaflet given |
| 102490 | 66Av.00 | Diabetic assessment of erectile dysfunction |
| 102611 | 66At111 | Type 2 diabetic dietary review |
| 102704 | 66At000 | Type I diabetic dietary review |
| 102767 | 67IJ100 | Pre-conception advice for diabetes mellitus |
| 103743 | 8IE2.00 | Diabetes care plan declined |
| 103798 | 9b92000 | Diabetic medicine |
| 103902 | C10FG11 | Type II diabetes mellitus with arthropathy |
| 103935 | 1IA..00 | No evidence of diabetic nephropathy |
| 104287 | 8Hlc.00 | Referral to community diabetes service |
| 104323 | C10F511 | Type II diabetes mellitus with gangrene |
| 104374 | 67D8.00 | Provision of diabetes clinical summary |
| 104453 | 66At011 | Type 1 diabetic dietary review |
| 104588 | 66Ay.00 | Gestational diabetes mellitus annual review |
| 104639 | C10FF11 | Type II diabetes mellitus with peripheral angiopathy |
| 105207 | 8HTE100 | Referral to community diabetes clinic |
| 105302 | K08yA00 | Proteinuric diabetic nephropathy |
| 105585 | 8CMW700 | Diabetes clinical pathway |
| 105740 | 2G5d.00 | O/E - Left diabetic foot at increased risk |
| 105741 | 2G5e.00 | O/E - Right diabetic foot at increased risk |
| 105784 | C109912 | Type 2 diabetes mellitus without complication |
| 105937 | 8IEQ.00 | Referral to community diabetes specialist nurse declined |
| 106061 | C10FP11 | Type II diabetes mellitus with ketoacidotic coma |
| 106218 | 9m0A.00 | Declined diabetic retinopathy screening |
| 106269 | 9m0..00 | Diabetic retinopathy screening administrative status |
| 106327 | 9m04.00 | Excluded from diabetic retinopathy screening |
| 106329 | 9m08.00 | Excluded from diabetic retinopathy screening as blind |
| 106332 | 9m00.00 | Eligible for diabetic retinopathy screening |
| 106350 | 9m05.00 | Excluded from diabetic retinopathy screening as moved away |
| 106352 | 9m06.00 | Excluded from diabetic retinopathy screening as deceased |
| 106360 | K27y700 | Erectile dysfunction due to diabetes mellitus |
| 106441 | 9m01.00 | Ineligible for diabetic retinopathy screening |
| 106528 | C10FN11 | Type II diabetes mellitus with ketoacidosis |
| 106604 | C11y500 | Pre-diabetes |
| 106622 | 38Gj.00 | QDiabetes risk calculator |
| 106679 | 8OA3.00 | Provision of written information about diabetes and driving |
| 106722 | 9Oy0300 | Diabetic foot screening invitation second letter |
| 106723 | 9Oy0200 | Diabetic foot screening invitation first letter |
| 106738 | 9Oy0000 | Diabetic foot screening invitation |
| 106953 | 8IEa.00 | Referral to DAFNE diabetes structured educn prog declined |
| 107361 | 679L200 | Education about diabetes and driving |
| 107414 | 8I94.00 | Diabetes structured education programme not available |
| 107423 | 661N400 | Diabetes self-management plan review |
| 107452 | 66o..00 | Further diabetic monitoring |
| 107464 | 66AS000 | Diabetes Year of Care annual review |
| 107508 | 66AH200 | Conversion to insulin by diabetes specialist nurse |
| 107554 | 38Gv.00 | Diabetes UK diabetes risk score |
| 107560 | 67H9.00 | Education about lifestyle for risk of diabetes |
| 107603 | C10P.00 | Diabetes mellitus in remission |
| 107701 | C10FK11 | Hyperosmolar non-ketotic state in type II diabetes mellitus |
| 107739 | 679L211 | Advice about diabetes and driving |
| 107793 | 9Oy0400 | Diabetic foot screening invitation third letter |
| 107824 | C10P100 | Type II diabetes mellitus in remission |
| 107881 | K08yA11 | Clinical diabetic nephropathy |
| 108005 | C109312 | Type 2 diabetes mellitus with multiple complications |
| 108013 | ZC2CB00 | Dietary advice for gestational diabetes |
| 108634 | 9NJy.00 | In-house diabetic foot screening |
| 108993 | 661M400 | Diabetes self-management plan agreed |
| 109103 | C109911 | Type II diabetes mellitus without complication |
| 109133 | L180700 | Pre-existing malnutrition-related diabetes mellitus |
| 109197 | C10FH11 | Type II diabetes mellitus with neuropathic arthropathy |
| Type I diabetes codes | | |
| 18683 | C10E500 | Type 1 diabetes mellitus with ulcer |
| 6509 | C108700 | Insulin dependent diabetes mellitus with retinopathy |
| 97446 | C108912 | Type 1 diabetes mellitus maturity onset |
| 30323 | C10EK00 | Type 1 diabetes mellitus with persistent proteinuria |
| 6791 | C108800 | Insulin dependent diabetes mellitus - poor control |
| 93468 | C10EG00 | Type 1 diabetes mellitus with peripheral angiopathy |
| 44440 | C108E00 | Insulin dependent diabetes mellitus with hypoglycaemic coma |
| 18230 | C108J12 | Type 1 diabetes mellitus with neuropathic arthropathy |
| 93878 | C10E511 | Type I diabetes mellitus with ulcer |
| 95992 | C108A11 | Type I diabetes mellitus without complication |
| 1038 | C100011 | Insulin dependent diabetes mellitus |
| 99311 | C10E111 | Type I diabetes mellitus with ophthalmic complications |
| 42831 | C10E200 | Type 1 diabetes mellitus with neurological complications |
| 35288 | C10E800 | Type 1 diabetes mellitus - poor control |
| 46301 | C10EC00 | Type 1 diabetes mellitus with polyneuropathy |
| 57621 | C108D00 | Insulin dependent diabetes mellitus with nephropathy |
| 49949 | C10E411 | Unstable type I diabetes mellitus |
| 66145 | C10EN11 | Type I diabetes mellitus with ketoacidotic coma |
| 22871 | C10EP00 | Type 1 diabetes mellitus with exudative maculopathy |
| 55239 | C10EQ00 | Type 1 diabetes mellitus with gastroparesis |
| 97894 | C10EP11 | Type I diabetes mellitus with exudative maculopathy |
| 98704 | C10E512 | Insulin dependent diabetes mellitus with ulcer |
| 40837 | C10EN00 | Type 1 diabetes mellitus with ketoacidotic coma |
| 24423 | C108.13 | Type I diabetes mellitus |
| 99719 | C10EA12 | Insulin-dependent diabetes without complication |
| 49554 | C10EF00 | Type 1 diabetes mellitus with diabetic cataract |
| 45914 | C108812 | Type 1 diabetes mellitus - poor control |
| 91942 | C10E311 | Type I diabetes mellitus with multiple complications |
| 47650 | C10E300 | Type 1 diabetes mellitus with multiple complications |
| 108360 | C10P000 | Type I diabetes mellitus in remission |
| 101311 | C10EC12 | Insulin dependent diabetes mellitus with polyneuropathy |
| 54008 | C10EJ00 | Type 1 diabetes mellitus with neuropathic arthropathy |
| 39070 | C10EE00 | Type 1 diabetes mellitus with hypoglycaemic coma |
| 40682 | C10E900 | Type 1 diabetes mellitus maturity onset |
| 109051 | C10E612 | Insulin dependent diabetes mellitus with gangrene |
| 45276 | C10E312 | Insulin dependent diabetes mellitus with multiple complicat |
| 101735 | C10E212 | Insulin-dependent diabetes mellitus with neurological comps |
| 60499 | C108600 | Insulin dependent diabetes mellitus with gangrene |
| 97474 | C108412 | Unstable type 1 diabetes mellitus |
| 26855 | C108400 | Unstable insulin dependent diabetes mellitus |
| 61829 | C108212 | Type 1 diabetes mellitus with neurological complications |
| 95343 | C10E711 | Type I diabetes mellitus with retinopathy |
| 102163 | C10ED12 | Insulin dependent diabetes mellitus with nephropathy |
| 51261 | C10E.12 | Insulin dependent diabetes mellitus |
| 97849 | C10E912 | Insulin dependent diabetes maturity onset |
| 18505 | C108.11 | IDDM-Insulin dependent diabetes mellitus |
| 1549 | C10E.00 | Type 1 diabetes mellitus |
| 69993 | C10E600 | Type 1 diabetes mellitus with gangrene |
| 62613 | C10EA11 | Type I diabetes mellitus without complication |
| 99716 | C10EE12 | Insulin dependent diabetes mellitus with hypoglycaemic coma |
| 60107 | C108411 | Unstable type I diabetes mellitus |
| 108724 | C10EQ11 | Type I diabetes mellitus with gastroparesis |
| 61344 | C108011 | Type I diabetes mellitus with renal complications |
| 69676 | C10EA00 | Type 1 diabetes mellitus without complication |
| 1647 | C108.00 | Insulin dependent diabetes mellitus |
| 102112 | C10E611 | Type I diabetes mellitus with gangrene |
| 72702 | C10E812 | Insulin dependent diabetes mellitus - poor control |
| 49146 | C108211 | Type I diabetes mellitus with neurological complications |
| 43921 | C10E400 | Unstable type 1 diabetes mellitus |
| 62352 | C108H11 | Type I diabetes mellitus with arthropathy |
| 41049 | C108712 | Type 1 diabetes mellitus with retinopathy |
| 68105 | C10EB00 | Type 1 diabetes mellitus with mononeuropathy |
| 102740 | C108112 | Type 1 diabetes mellitus with ophthalmic complications |
| 65616 | C108H00 | Insulin dependent diabetes mellitus with arthropathy |
| 60208 | C108J11 | Type I diabetes mellitus with neuropathic arthropathy |
| 44443 | C108500 | Insulin dependent diabetes mellitus with ulcer |
| 46850 | C108811 | Type I diabetes mellitus - poor control |
| 18387 | C10E700 | Type 1 diabetes mellitus with retinopathy |
| 10692 | C10EM00 | Type 1 diabetes mellitus with ketoacidosis |
| 41716 | C108C00 | Insulin dependent diabetes mellitus with polyneuropathy |
| 69043 | ZC2C900 | Dietary advice for type I diabetes |
| 102946 | C10E012 | Insulin-dependent diabetes mellitus with renal complications |
| 42729 | C108E11 | Type I diabetes mellitus with hypoglycaemic coma |
| 54600 | C10E412 | Unstable insulin dependent diabetes mellitus |
| 31310 | C108900 | Insulin dependent diabetes maturity onset |
| 96235 | C10E911 | Type I diabetes mellitus maturity onset |
| 10418 | C10ED00 | Type 1 diabetes mellitus with nephropathy |
| 52283 | C108200 | Insulin-dependent diabetes mellitus with neurological comps |
| 46963 | C108000 | Insulin-dependent diabetes mellitus with renal complications |
| 66872 | C108D11 | Type I diabetes mellitus with nephropathy |
| 24694 | C108B00 | Insulin dependent diabetes mellitus with mononeuropathy |
| 17858 | C108.12 | Type 1 diabetes mellitus |
| 56448 | C108A00 | Insulin-dependent diabetes without complication |
| 47582 | C10E000 | Type 1 diabetes mellitus with renal complications |
| 30294 | C10EL00 | Type 1 diabetes mellitus with persistent microalbuminuria |
| 52104 | C108300 | Insulin dependent diabetes mellitus with multiple complicatn |
| 63017 | C108911 | Type I diabetes mellitus maturity onset |
| 62209 | C10EM11 | Type I diabetes mellitus with ketoacidosis |
| 91943 | C10EC11 | Type I diabetes mellitus with polyneuropathy |
| 17545 | C108F11 | Type I diabetes mellitus with diabetic cataract |
| 68390 | C108512 | Type 1 diabetes mellitus with ulcer |
| 105337 | C10E811 | Type I diabetes mellitus - poor control |
| 47649 | C10E100 | Type 1 diabetes mellitus with ophthalmic complications |
| 2478 | 66AJ100 | Brittle diabetes |
| 70766 | C108E12 | Type 1 diabetes mellitus with hypoglycaemic coma |
| 100770 | C10EF12 | Insulin dependent diabetes mellitus with diabetic cataract |
| 38161 | C108711 | Type I diabetes mellitus with retinopathy |
| 108007 | C108311 | Type I diabetes mellitus with multiple complications |
| 51957 | C108511 | Type I diabetes mellitus with ulcer |
| 99231 | C108B11 | Type I diabetes mellitus with mononeuropathy |
| 102620 | C10EL11 | Type I diabetes mellitus with persistent microalbuminuria |
| 44260 | C108F00 | Insulin dependent diabetes mellitus with diabetic cataract |
| 49276 | C108100 | Insulin-dependent diabetes mellitus with ophthalmic comps |
| 18642 | C10EH00 | Type 1 diabetes mellitus with arthropathy |
| 32359 | ZRbH.00 | Perceived control of insulin-dependent diabetes |
| 98071 | C10E112 | Insulin-dependent diabetes mellitus with ophthalmic comps |
| 12455 | C10E.11 | Type I diabetes mellitus |
| 21983 | C108012 | Type 1 diabetes mellitus with renal complications |
| 93875 | C10E712 | Insulin dependent diabetes mellitus with retinopathy |
| Under- and Over-active thyroid disease codes | | |
| 4937 | 143..11 | H/O: thyroid disorder |
| 6245 | 1431.00 | H/O: hyperthyroidism |
| 8038 | 1431.11 | H/O: thyrotoxicosis |
| 3611 | 1432.00 | H/O: hypothyroidism |
| 35608 | 1433.00 | H/O: thyroid disorder NOS |
| 104582 | 1IC..00 | Congenital hypothyroidism not suspected |
| 102442 | 1JM..00 | Suspected hypothyroidism |
| 108482 | 1JM0.00 | Suspected congenital hypothyroidism |
| 26362 | 212P.00 | Hyperthyroidism resolved |
| 8588 | 22H..00 | O/E - thyroid gland |
| 36039 | 22H1.00 | O/E - thyroid gland - NOS |
| 15728 | 22H2.00 | O/E - thyroid swelling -unilat |
| 16057 | 22H3.00 | O/E - thyroid swelling -bilat. |
| 728 | 22H4.00 | O/E - thyroid lump |
| 42910 | 22HZ.00 | O/E - thyroid gland NOS |
| 3789 | 43G5.00 | Thyroid autoantibodies |
| 27807 | 442C.00 | Thyroid horm tests borderline |
| 20970 | 442G.00 | Thyroid hormone tests abnormal |
| 18598 | 442I.00 | Thyroid function tests abnormal |
| 13906 | 442J.00 | Thyroid function test |
| 3622 | 5A11.00 | Thyroid gland ablat - irradiat |
| 67248 | 5A12.00 | Thyroid tumour/metast irradiat |
| 8268 | 66B..00 | Thyroid disease monitoring |
| 31184 | 66B2.00 | Follow-up thyroid assessment |
| 38459 | 66B3.00 | Inactive thyroid disease |
| 28530 | 66B4.00 | Thyroid eye disease |
| 30389 | 66B5.00 | Thyroid symptom change |
| 57992 | 66B6.00 | Thyroid drug side effects |
| 58544 | 66B7.00 | Thyroid-dubious diagn.criteria |
| 24681 | 66B8.00 | Thyroid dis.treatment changed |
| 38292 | 66B9.00 | Thyroid dis.treatment started |
| 40389 | 66BA.00 | Thyroid dis.treatment stopped |
| 95885 | 66BB.00 | Hypothyroidism annual review |
| 28822 | 66BZ.00 | Thyroid disease monitoring NOS |
| 9128 | 711..12 | Thyroid gland operations |
| 7712 | 7110.00 | Thyroidectomy operations |
| 25323 | 7110.11 | Excision of thyroid gland operations |
| 8384 | 7110000 | Total thyroidectomy |
| 2320 | 7110100 | Subtotal thyroidectomy |
| 16784 | 7110111 | Bilateral subtotal thyroidectomy |
| 5774 | 7110200 | Hemithyroidectomy |
| 3656 | 7110300 | Lobectomy of thyroid gland NEC |
| 34101 | 7110400 | Isthmectomy of thyroid gland |
| 810 | 7110500 | Partial thyroidectomy NEC |
| 34289 | 7110600 | Thyroidectomy NEC |
| 38650 | 7110y00 | Other specified thyroidectomy |
| 2039 | 7110z00 | Thyroidectomy NOS |
| 59434 | 7111.00 | Operations on aberrant thyroid tissue |
| 50209 | 7111000 | Excision of substernal thyroid tissue |
| 57497 | 7111100 | Excision of sublingual thyroid tissue |
| 93212 | 7111y00 | Other specified operation on aberrant thyroid tissue |
| 105906 | 7111z00 | Operation on aberrant thyroid tissue NOS |
| 16027 | 7113.00 | Other operations on thyroid gland |
| 2592 | 7113000 | Excision of lesion of thyroid gland |
| 23090 | 7113100 | Biopsy of lesion of thyroid gland |
| 62125 | 7113200 | Incision of lesion of thyroid gland |
| 9893 | 7113300 | Exploration of thyroid gland |
| 38303 | 7113400 | Biopsy of thyroid gland |
| 61197 | 7113y00 | Other specified other operation on thyroid gland |
| 40542 | 7113z00 | Other operation on thyroid gland NOS |
| 86329 | 7L1Z400 | Oral delivery of radiotherapy for thyroid ablation |
| 7541 | 7N10000 | [SO]Thyroid gland |
| 45075 | 7N10100 | [SO]Aberrant thyroid tissue |
| 103838 | 8BPG.00 | Thyroid stimulating hormone suppression therapy |
| 85955 | 8CR5.00 | Hypothyroidism clinical management plan |
| 62116 | 9N4T.00 | DNA - Did not attend hyperthyroidism clinic |
| 28735 | 9Oj..00 | Hypothyroidism monitoring administration |
| 46057 | 9Oj0.00 | Hypothyroidism monitoring first letter |
| 46630 | 9Oj1.00 | Hypothyroidism monitoring second letter |
| 46640 | 9Oj2.00 | Hypothyroidism monitoring third letter |
| 19367 | 9Oj3.00 | Hypothyroidism monitoring verbal invite |
| 85661 | 9Oj4.00 | Hypothyroidism monitoring telephone invitation |
| 28681 | 9h7..00 | Exception reporting: thyroid quality indicators |
| 11685 | 9h71.00 | Excepted from thyroid quality indicators: Patient unsuitable |
| 25917 | 9h72.00 | Excepted from thyroid quality indicators: Informed dissent |
| 5637 | B53..00 | Malignant neoplasm of thyroid gland |
| 37758 | B7G..00 | Benign neoplasm of thyroid gland |
| 2610 | B7G..11 | Adenoma of thyroid gland |
| 8958 | B8yy000 | Carcinoma in situ of thyroid gland |
| 17415 | B924000 | Neoplasm of uncertain behaviour of thyroid gland |
| 19263 | BB5f.00 | [M]Thyroid adenoma and adenocarcinoma |
| 38685 | BB5fz00 | [M]Thyroid adenoma or adenocarcinoma NOS |
| 40608 | ByuB.00 | [X]Malignant neoplasm of thyroid and other endocrine glands |
| 1882 | C0...00 | Disorders of thyroid gland |
| 3655 | C000.11 | Retrosternal thyroid goitre |
| 60288 | C000.12 | Substernal thyroid goitre |
| 2572 | C000.13 | Thyroid nodule |
| 1881 | C00z.11 | Thyroid enlargement |
| 677 | C02..00 | Thyrotoxicosis |
| 1472 | C02..11 | Hyperthyroidism |
| 100476 | C020200 | Thyroid-associated dermopathy |
| 49508 | C024.00 | Thyrotoxicosis from ectopic thyroid nodule |
| 64656 | C024000 | Thyrotoxicosis from ectopic thyroid nodule with no crisis |
| 56270 | C024z00 | Thyrotoxicosis from ectopic thyroid nodule NOS |
| 106640 | C025.00 | Subclinical hyperthyroidism |
| 43136 | C02y.00 | Thyrotoxicosis of other specified origin |
| 51273 | C02y000 | Thyrotoxicosis of other specified origin with no crisis |
| 106532 | C02y100 | Thyrotoxicosis of other specified origin with crisis |
| 19205 | C02y300 | Thyroid crisis |
| 34220 | C02yz00 | Thyrotoxicosis of other specified origin NOS |
| 15565 | C02z.00 | Thyrotoxicosis without mention of goitre or other cause |
| 26701 | C02z000 | Thyrotoxicosis without mention of goitre or cause no crisis |
| 3194 | C02z100 | Thyrotoxicosis without mention of goitre, cause with crisis |
| 26699 | C02zz00 | Thyrotoxicosis NOS |
| 10097 | C03..00 | Congenital hypothyroidism |
| 69290 | C03y.00 | Other specified congenital hypothyroidism |
| 31612 | C03y000 | Congenital hypothyroidism with diffuse goitre |
| 93159 | C03y100 | Congenital hypothyroidism without goitre |
| 51481 | C03z.00 | Congenital hypothyroidism NOS |
| 93323 | C03z.11 | Congenital thyroid insufficiency |
| 3290 | C04..00 | Acquired hypothyroidism |
| 14704 | C04..12 | Thyroid deficiency |
| 273 | C04..13 | Hypothyroidism |
| 28852 | C040.00 | Postsurgical hypothyroidism |
| 47521 | C040.11 | Post ablative hypothyroidism |
| 50275 | C041.00 | Other postablative hypothyroidism |
| 11322 | C041000 | Irradiation hypothyroidism |
| 51706 | C041z00 | Postablative hypothyroidism NOS |
| 34221 | C042.00 | Iodine hypothyroidism |
| 25913 | C043.00 | Other iatrogenic hypothyroidism |
| 15743 | C043000 | Hypothyroidism resulting from para-aminosalicylic acid |
| 97090 | C043100 | Hypothyroidism resulting from phenylbutazone |
| 94915 | C043200 | Hypothyroidism resulting from resorcinol |
| 38976 | C043z00 | Iatrogenic hypothyroidism NOS |
| 50860 | C044.00 | Postinfectious hypothyroidism |
| 46345 | C045.00 | Acquired atrophy of thyroid |
| 95830 | C047.00 | Subclinical hypothyroidism |
| 24748 | C04y.00 | Other acquired hypothyroidism |
| 3941 | C04z.00 | Hypothyroidism NOS |
| 20310 | C04z.11 | Pretibial myxoedema - hypothyroid |
| 23014 | C04z.12 | Thyroid insufficiency |
| 18282 | C04z.13 | Hypothyroid goitre, acquired |
| 56722 | C04z000 | Premature puberty due to hypothyroidism |
| 1346 | C05..00 | Thyroiditis |
| 4898 | C050.00 | Acute thyroiditis |
| 67972 | C050000 | Acute nonsuppurative thyroiditis |
| 42323 | C050z00 | Acute thyroiditis NOS |
| 30799 | C051.00 | Subacute thyroiditis |
| 21747 | C051.11 | De Quervain's thyroiditis |
| 26833 | C052.00 | Chronic lymphocytic thyroiditis |
| 3857 | C052.11 | Autoimmune thyroiditis |
| 70244 | C053.00 | Chronic fibrous thyroiditis |
| 53667 | C053.11 | Riedel's thyroiditis |
| 61026 | C054.00 | Iatrogenic thyroiditis |
| 65444 | C05y.00 | Other and unspecified chronic thyroiditis |
| 65907 | C05y400 | Chronic thyroiditis with transient thyrotoxicosis |
| 20909 | C05z.00 | Thyroiditis NOS |
| 43871 | C06..00 | Other disorders of thyroid |
| 43245 | C06y.00 | Other specified thyroid disorders |
| 41014 | C06y000 | Thyroid-binding globulin abnormality |
| 69113 | C06y100 | Thyroid atrophy |
| 27996 | C06yz00 | Other specified thyroid disorder NOS |
| 35957 | C06z.00 | Thyroid disorder NOS |
| 718 | C0A5.00 | Subclinical iodine-deficiency hypothyroidism |
| 11146 | C134300 | TSH - thyroid-stimulating hormone deficiency |
| 65175 | Cyu1.00 | [X]Disorders of thyroid gland |
| 73107 | Cyu1100 | [X]Other sp cified hypothyroidism |
| 72690 | Cyu1300 | [X]Other thyrotoxicosis |
| 95335 | Cyu1400 | [X]Other chronic thyroiditis |
| 73096 | Cyu1500 | [X]Other specified disorders of thyroid |
| 61069 | F381400 | Myasthenic syndrome due to hypothyroidism |
| 47695 | F381600 | Myasthenic syndrome due to thyrotoxicosis |
| 48167 | F395400 | Myopathy due to thyrotoxicosis |
| 68626 | FyuBD00 | [X]Dysthyroid exophthalmos |
| 11947 | L181500 | Postpartum thyroiditis |
| 10638 | PK25.00 | Anomalies of thyroid gland NEC |
| 39281 | PK25000 | Aberrant thyroid gland |
| 10704 | PK25011 | Retrosternal thyroid gland |
| 3374 | PK25100 | Congenital absence of thyroid gland |
| 70581 | PK25z00 | Anomaly of thyroid gland NEC NOS |
| 58833 | Q433700 | Neonatal jaundice with congenital hypothyroidism |
| 48010 | Q443.00 | Neonatal thyrotoxicosis |
| 1658 | R145.00 | [D]Thyroid function test abnormal |
| 27639 | R145000 | [D]Thyroid scan abnormal |
| 51339 | R145100 | [D]Thyroid uptake abnormal |
| 48045 | R145z00 | [D]Thyroid function tests abnormal NOS |
| 72360 | TJ27.00 | Adverse reaction to thyroid and thyroid derivatives |
| 96618 | TJ27z00 | Adverse reaction to thyroid and thyroid derivatives NOS |
| 53536 | TJ28.00 | Adverse reaction to antithyroid agents |
| 104753 | TJ28z00 | Adverse reaction to antithyroid agents NOS |
| 45916 | U602100 | [X]Thyroid horms + substits caus adverse eff in therap use |
| 72206 | U602200 | [X]Antithyroid drugs caus adverse effects in therapeut use |
| 59923 | U602211 | [X] Adverse reaction to antithyroid agents |
| 35771 | ZV10y15 | [V]Personal history of malignant neoplasm of thyroid |
| Rheumatoid Arthritis codes | | |
| 6639 | 14G1.00 | H/O: rheumatoid arthritis |
| 100187 | 38DZ.00 | Disease activity score in rheumatoid arthritis |
| 17412 | 66H..13 | Rheumatoid arthrit. monitoring |
| 102088 | 7P20300 | Delivery of rehabilitation for rheumatoid arthritis |
| 62401 | F371200 | Polyneuropathy in rheumatoid arthritis |
| 31209 | F396400 | Myopathy due to rheumatoid arthritis |
| 49787 | G5y8.00 | Rheumatoid myocarditis |
| 43816 | G5yA.00 | Rheumatoid carditis |
| 9954 | H570.00 | Rheumatoid lung |
| 27603 | N04..00 | Rheumatoid arthritis and other inflammatory polyarthropathy |
| 844 | N040.00 | Rheumatoid arthritis |
| 44743 | N040000 | Rheumatoid arthritis of cervical spine |
| 44203 | N040100 | Other rheumatoid arthritis of spine |
| 21358 | N040200 | Rheumatoid arthritis of shoulder |
| 100914 | N040400 | Rheumatoid arthritis of acromioclavicular joint |
| 59738 | N040500 | Rheumatoid arthritis of elbow |
| 63365 | N040600 | Rheumatoid arthritis of distal radio-ulnar joint |
| 48832 | N040700 | Rheumatoid arthritis of wrist |
| 42299 | N040800 | Rheumatoid arthritis of MCP joint |
| 41941 | N040900 | Rheumatoid arthritis of PIP joint of finger |
| 63198 | N040A00 | Rheumatoid arthritis of DIP joint of finger |
| 49067 | N040B00 | Rheumatoid arthritis of hip |
| 100776 | N040C00 | Rheumatoid arthritis of sacro-iliac joint |
| 50863 | N040D00 | Rheumatoid arthritis of knee |
| 51239 | N040F00 | Rheumatoid arthritis of ankle |
| 73619 | N040G00 | Rheumatoid arthritis of subtalar joint |
| 70658 | N040H00 | Rheumatoid arthritis of talonavicular joint |
| 71784 | N040J00 | Rheumatoid arthritis of other tarsal joint |
| 51238 | N040K00 | Rheumatoid arthritis of 1st MTP joint |
| 99414 | N040L00 | Rheumatoid arthritis of lesser MTP joint |
| 30548 | N040N00 | Rheumatoid vasculitis |
| 6916 | N040P00 | Seronegative rheumatoid arthritis |
| 53621 | N040R00 | Rheumatoid nodule |
| 31054 | N040S00 | Rheumatoid arthritis - multiple joint |
| 8350 | N040T00 | Flare of rheumatoid arthritis |
| 23552 | N041.00 | Felty's syndrome |
| 49227 | N042.00 | Other rheumatoid arthropathy + visceral/systemic involvement |
| 46436 | N042100 | Rheumatoid lung disease |
| 5723 | N042200 | Rheumatoid nodule |
| 37431 | N042z00 | Rheumatoid arthropathy + visceral/systemic involvement NOS |
| 50644 | N043000 | Juvenile rheumatoid arthropathy unspecified |
| 47831 | N043100 | Acute polyarticular juvenile rheumatoid arthritis |
| 21533 | N043200 | Pauciarticular juvenile rheumatoid arthritis |
| 36276 | N043300 | Monarticular juvenile rheumatoid arthritis |
| 27557 | N043z00 | Juvenile rheumatoid arthritis NOS |
| 31360 | N045500 | Juvenile rheumatoid arthritis |
| 9707 | N047.00 | Seropositive errosive rheumatoid arthritis |
| 12019 | N04X.00 | Seropositive rheumatoid arthritis, unspecified |
| 31724 | N04y000 | Rheumatoid lung |
| 56838 | N04y011 | Caplan's syndrome |
| 28853 | N04y012 | Fibrosing alveolitis associated with rheumatoid arthritis |
| 93715 | Nyu1100 | [X]Other seropositive rheumatoid arthritis |
| 70221 | Nyu1200 | [X]Other specified rheumatoid arthritis |
| 56202 | Nyu1G00 | [X]Seropositive rheumatoid arthritis, unspecified |
| Inflammatory Bowel Disease codes | | |
| 12575 | N045300 | Juvenile arthritis in Crohn's disease |
| 43090 | J41yz00 | Other idiopathic proctocolitis NOS |
| 39037 | J401200 | Exacerbation of Crohn's disease of large intestine |
| 63036 | J400100 | Regional enteritis of the jejunum |
| 62628 | J401000 | Regional enteritis of the colon |
| 69959 | Jyu4000 | [X]Other Crohn's disease |
| 11337 | ZR3S.00 | Crohn's disease activity index |
| 1784 | J41..12 | Ulcerative colitis and/or proctitis |
| 22516 | J410400 | Exacerbation of ulcerative colitis |
| 6650 | J410.00 | Ulcerative proctocolitis |
| 51578 | J40..12 | Granulomatous enteritis |
| 64773 | J401100 | Regional enteritis of the rectum |
| 5133 | J41..00 | Idiopathic proctocolitis |
| 59994 | J40z.11 | Crohn's disease NOS |
| 33456 | J410z00 | Ulcerative proctocolitis NOS |
| 52449 | J40z.00 | Regional enteritis NOS |
| 24550 | J41y.00 | Other idiopathic proctocolitis |
| 51576 | J400.00 | Regional enteritis of the small bowel |
| 20480 | N031100 | Arthropathy in Crohn's disease |
| 66238 | J400300 | Crohn's disease of the ileum unspecified |
| 71945 | J400000 | Regional enteritis of the duodenum |
| 11286 | J40..00 | Regional enteritis - Crohn's disease |
| 107313 | 8Cc5.00 | Management of inflammatory bowel disease |
| 28476 | J400200 | Crohn's disease of the terminal ileum |
| 53743 | Jyu4100 | [X]Other ulcerative colitis |
| 96976 | J4z6.00 | Indeterminate colitis |
| 5749 | 14C4.11 | H/O: ulcerative colitis |
| 11119 | ZR3S.11 | CDAI - Crohn's disease activity index |
| 39278 | J400400 | Crohn's disease of the ileum NOS |
| 6538 | J401z11 | Crohn's colitis |
| 44426 | J401.00 | Regional enteritis of the large bowel |
| 39119 | J436000 | Collagenous colitis |
| 36913 | J400500 | Exacerbation of Crohn's disease of small intestine |
| 30678 | J436.00 | Microscopic colitis |
| 15207 | J41z.00 | Idiopathic proctocolitis NOS |
| 1796 | J4...12 | Inflammatory bowel disease |
| 9359 | J400z00 | Crohn's disease of the small bowel NOS |
| 35424 | J436100 | Lymphocytic colitis |
| 20688 | J401z00 | Crohn's disease of the large bowel NOS |
| 17641 | N031000 | Arthropathy in ulcerative colitis |
| 704 | J410100 | Ulcerative colitis |
| 71083 | N045400 | Juvenile arthritis in ulcerative colitis |
| 593 | J40..11 | Crohn's disease |
| 29616 | J08z900 | Orofacial Crohn's disease |
| Coeliac disease codes | | |
| 44310 | J690z00 | Coeliac disease NOS |
| 102617 | 6648000 | Coeliac disease annual review |
| 98297 | 68W4.00 | Coeliac disease autoantibody profile positive |
| 1515 | J690.00 | Coeliac disease |
| 102315 | 6648.00 | Coeliac disease monitoring |
| 106326 | 9mB1.00 | Coeliac disease monitoring invitation first letter |
| 63195 | J690100 | Acquired coeliac disease |
| 106282 | 9mB..00 | Coeliac disease monitoring invitation |
| 103379 | 8IAp.00 | Coeliac disease annual review declined |
| 107845 | 13YB.00 | Coeliac UK member |
| 45925 | ZC2C200 | Dietary advice for coeliac disease |
| 68680 | J690.11 | Coeliac rickets |
| 62397 | J690000 | Congenital coeliac disease |
| Vitiligo Codes | |  |
| 975 | M295100 | Vitiligo |
| 43328 | F4E5311 | Vitiligo of eyelid |
| Addision's disease codes | | |
| 12227 | C154600 | Addisonian crisis |
| 4481 | C154100 | Addison's disease |
| 2813 | D010.11 | Addison's anaemia |
| 4042 | C154011 | Addisonian crisis |
| 43631 | M210.11 | Addison's keloid |
| 69198 | F395000 | Myopathy due to Addison's disease |
| Chronic Obstructive Pulmonary Disease codes | | |
| 10802 | H37..00 | Moderate chronic obstructive pulmonary disease |
| 54893 | H582.00 | Compensatory emphysema |
| 33450 | H32z.00 | Emphysema NOS |
| 15157 | H31z.00 | Chronic bronchitis NOS |
| 40788 | H32y.00 | Other emphysema |
| 56126 | Q312.00 | Perinatal interstitial emphysema and related conditions |
| 60188 | H320200 | Giant bullous emphysema |
| 103758 | 8Hkw.00 | Referral to COPD community nursing team |
| 14798 | H312100 | Emphysematous bronchitis |
| 92955 | H32y000 | Acute vesicular emphysema |
| 99948 | 9kf0.00 | COPD patient unsuitable for pulmonary rehab - enh serv admin |
| 3113 | C154.00 | Corticoadrenal insufficiency |
| 93568 | H39..00 | Very severe chronic obstructive pulmonary disease |
| 37371 | 66YD.00 | Chronic obstructive pulmonary disease monitoring due |
| 46036 | 66Yi.00 | Multiple COPD emergency hospital admissions |
| 40159 | H311000 | Purulent chronic bronchitis |
| 18621 | 66YL.00 | Chronic obstructive pulmonary disease follow-up |
| 64721 | H464000 | Chronic emphysema due to chemical fumes |
| 102685 | 66YB000 | Chronic obstructive pulmonary disease 3 monthly review |
| 107877 | 8IEy.00 | Chronic obstructive pulmon dis wr self managem plan declined |
| 103558 | 8CeD.00 | Preferred place of care for next exacerbation of COPD |
| 57010 | Q312300 | Perinatal interstitial emphysema |
| 104710 | 9NgP.11 | On COPD (chr obstruc pulmonary disease) supportv cre pathway |
| 104169 | 661N300 | COPD self-management plan review |
| 11019 | 8H2R.00 | Admit COPD emergency |
| 37959 | H311100 | Fetid chronic bronchitis |
| 44962 | C153.00 | Other corticoadrenal overactivity |
| 9876 | H38..00 | Severe chronic obstructive pulmonary disease |
| 37247 | H3z..11 | Chronic obstructive pulmonary disease NOS |
| 11287 | 66YM.00 | Chronic obstructive pulmonary disease annual review |
| 19434 | 1J71.00 | Suspected chronic obstructive pulmonary disease |
| 11266 | 9h52.00 | Excepted from COPD quality indicators: Informed dissent |
| 97800 | 9kf..00 | COPD - enhanced services administration |
| 11026 | 9h51.00 | Excepted from COPD quality indicators: Patient unsuitable |
| 1001 | H3...00 | Chronic obstructive pulmonary disease |
| 25603 | H310.00 | Simple chronic bronchitis |
| 68662 | H320100 | Zonal bullous emphysema |
| 99536 | H320300 | Bullous emphysema with collapse |
| 23492 | H320z00 | Chronic bullous emphysema NOS |
| 10980 | H322.00 | Centrilobular emphysema |
| 104608 | H3A..00 | End stage chronic obstructive airways disease |
| 16410 | H32yz00 | Other emphysema NOS |
| 22905 | H581.00 | Interstitial emphysema |
| 104481 | 8CMV.00 | Has chronic obstructive pulmonary disease care plan |
| 104117 | 661M300 | COPD self-management plan agreed |
| 45771 | 66Yh.00 | Chronic obstructive pulmonary disease does not disturb sleep |
| 18501 | 66YI.00 | COPD self-management plan given |
| 47236 | ZV7B311 | [V]Screening for chronic bronchitis |
| 19106 | 66Yd.00 | COPD accident and emergency attendance since last visit |
| 45998 | 66YT.00 | Chronic obstructive pulmonary disease monitoring by doctor |
| 103494 | 14B3.12 | History of chronic obstructive pulmonary disease |
| 61118 | H310z00 | Simple chronic bronchitis NOS |
| 98284 | 9kf1.00 | Refer COPD structured smoking assessment - enhanc serv admin |
| 67040 | H3y..11 | Other specified chronic obstructive pulmonary disease |
| 794 | H32..00 | Emphysema |
| 101042 | 8BMW.00 | Issue of chronic obstructive pulmonary disease rescue pack |
| 48483 | ZV7B312 | [V]Screening for emphysema |
| 5710 | H3z..00 | Chronic obstructive airways disease NOS |
| 18717 | 9h5..00 | Exception reporting: COPD quality indicators |
| 11150 | H311.00 | Mucopurulent chronic bronchitis |
| 19003 | 66Ye.00 | Emergency COPD admission since last appointment |
| 61513 | H311z00 | Mucopurulent chronic bronchitis NOS |
| 45777 | 8CR1.00 | Chronic obstructive pulmonary disease clini management plan |
| 56860 | H320000 | Segmental bullous emphysema |
| 27819 | H312.00 | Obstructive chronic bronchitis |
| 26018 | 66YS.00 | Chronic obstructive pulmonary disease monitoring by nurse |
| 103400 | 9kf1.11 | Referred for COPD structured smoking assessment |
| 66043 | H31y.00 | Other chronic bronchitis |
| 100877 | 38Dd.00 | Clinical chronic obstructive pulmonary disease questionnaire |
| 103864 | 9kf0.11 | COPD patient unsuitable for pulmonary rehabilitation |
| 12166 | H3y..00 | Other specified chronic obstructive airways disease |
| 26306 | H320.00 | Chronic bullous emphysema |
| 46578 | H321.00 | Panlobular emphysema |
| 42313 | 679V.00 | Health education - chronic obstructive pulmonary disease |
| 28743 | 66Yf.00 | Number of COPD exacerbations in past year |
| 59263 | H32y111 | Acute interstitial emphysema |
| 19428 | 1I70.00 | Chronic obstructive pulmonary disease excluded by spirometry |
| 105457 | 8CMW500 | Chronic obstructive pulmonary disease care pathway |
| 106945 | 8IEZ.00 | Chronic obstructive pulmonary disease rescue pack declined |
| 42624 | 66YL.12 | COAD follow-up |
| 68066 | H31yz00 | Other chronic bronchitis NOS |
| 103007 | 66YB100 | Chronic obstructive pulmonary disease 6 monthly review |
| 103760 | 9kf2.11 | COPD structured smoking assessment declined |
| 106637 | 9Nk7000 | Seen in chronic obstructive pulmonary disease clinic |
| 19721 | 8CE6.00 | Chronic obstructive pulmonary disease leaflet given |
| 28755 | 9Oi0.00 | Chronic obstructive pulmonary disease monitoring 1st letter |
| 34215 | 9Oi2.00 | Chronic obstructive pulmonary disease monitoring 3rd letter |
| 45770 | 66Yg.00 | Chronic obstructive pulmonary disease disturbs sleep |
| 12396 | C154z00 | Corticoadrenal insufficiency NOS |
| 998 | H3...11 | Chronic obstructive airways disease |
| 42258 | 9Oi3.00 | Chronic obstructive pulmonary disease monitoring verb invite |
| 9520 | 66YB.00 | Chronic obstructive pulmonary disease monitoring |
| 66058 | Hyu3000 | [X]Other emphysema |
| 62913 | ZV7B300 | [V]Screening for chronic bronchitis or emphysema |
| 44525 | H312z00 | Obstructive chronic bronchitis NOS |
| 3243 | H31..00 | Chronic bronchitis |
| 24248 | H313.00 | Mixed simple and mucopurulent chronic bronchitis |
| 63479 | H32y200 | MacLeod's unilateral emphysema |
| 10863 | H36..00 | Mild chronic obstructive pulmonary disease |
| 100237 | 38Dg.00 | Chronic obstructive pulmonary disease assessment test |
| 35303 | 9N4W.00 | DNA - Did not attend COPD clinic |
| 34202 | 9Oi1.00 | Chronic obstructive pulmonary disease monitoring 2nd letter |
| 18792 | 9Oi..00 | Chronic obstructive pulmonary disease monitoring admin |
| 18476 | 66YL.11 | COPD follow-up |
| 104265 | 9e03.00 | GP OOH service notified of COPD care plan |
| 1446 | H312200 | Acute exacerbation of chronic obstructive airways disease |
| 104985 | 9NgP.00 | On chronic obstructive pulmonary disease supprtv cre pathway |
| 65733 | Hyu3100 | [X]Other specified chronic obstructive pulmonary disease |
| 98283 | 9kf2.00 | COPD structured smoking assessment declined - enh serv admin |
| 38074 | 9Oi4.00 | Chronic obstructive pulmonary disease monitor phone invite |
| Alpha-1 antitrypsin deficiency codes | | |
| 27158 | 478F.00 | Faecal A1 antitrypsin |
| 100208 | 12a..00 | Family history of alpha-1-antitrypsin deficiency |
| 3019 | C376200 | Alpha-1-antitrypsin deficiency |
| 25589 | C376100 | Alpha-1-antitrypsin hepatitis |
| 27114 | 44N4.00 | Electrophoresis: alpha-1-glob. |
| 25760 | 4L00.00 | Alpha-1-antitrypsin phenotype |
| 99923 | 4L15.00 | Alpha-1-antitrypsin genotyping |
| 104416 | 4Q3M.00 | Alpha-1-antitrypsin level |
| 19898 | 44C6.00 | Serum A1 - antitrypsin |
| 63917 | 44Co.00 | Serum alpha-1-antichymotrypsin level |
| Pulmonary fibrosis codes | | |
| 106515 | Hyu4300 | [X]Hypersensitivity pneumonitis due to other organic dusts |
| 46795 | Q317100 | Prematurity with interstitial pulmonary fibrosis |
| 45948 | H470300 | Pneumonitis due to inhalation of vomitus |
| 46977 | H35z.00 | Allergic alveolitis and pneumonitis NOS |
| 103637 | H35..11 | Hypersensitivity pneumonitis |
| 49025 | H460100 | Acute pneumonitis due to chemical fumes |
| 7791 | H55..00 | Postinflammatory pulmonary fibrosis |
| 59083 | H470100 | Pneumonitis due to inhalation of gastric secretions |
| 94575 | H433.00 | Graphite fibrosis of lung |
| 94894 | H431.00 | Bauxite fibrosis of lung |
| 66104 | H470200 | Pneumonitis due to inhalation of milk |
| 103559 | H563300 | Usual interstitial pneumonitis |
| 26278 | H357.00 | 'Ventilation' pneumonitis |
| 50876 | H472.00 | Asp pneumonitis due to anaesthesia during labour and deliv |
| 66773 | H471z00 | Pneumonitis due to inhalation of oil or essence NOS |
| 30996 | H470211 | Milk inhalation pneumonitis |
| 55758 | H460z00 | Bronchitis and pneumonitis due to chemical fumes NOS |
| 56647 | H471.00 | Pneumonitis due to inhalation of oil or essence |
| 47782 | H464200 | Chronic pulmonary fibrosis due to chemical fumes |
| 2680 | 23E5.00 | O/E - fibrosis of lung present |
| 38639 | H460.00 | Bronchitis and pneumonitis due to chemical fumes |
| 18130 | H4y0000 | Acute radiation pneumonitis |
| 53095 | H35zz00 | Allergic alveolitis and pneumonitis NOS |
| 16741 | A114.00 | Tuberculous fibrosis of lung |
| 104915 | H58y700 | Interstitial lung disease due to connective tissue disease |
| 11833 | H35z100 | Hypersensitivity pneumonitis NOS |
| 3847 | H470.00 | Pneumonitis due to inhalation of food or vomitus |
| 10992 | H47..11 | Aspiration pneumonitis |
| 45072 | A785000 | Cytomegaloviral pneumonitis |
| 54252 | H47yz00 | Pneumonitis due to inhalation of solid or liquid NOS |
| 33837 | H470z00 | Pneumonitis due to inhalation of food or vomitus NOS |
| 7994 | 23E5.11 | O/E - fibrosis of lung |
| 51372 | Q311z11 | Pneumonitis due to fetal aspiration |
| 56385 | H470311 | Vomit inhalation pneumonitis |
| 8317 | H58y300 | Interstitial lung disease NEC |
| 65117 | A789900 | HIV disease resulting in lymphoid interstitial pneumonitis |
| 103472 | H563200 | Pulmonary fibrosis |
| 9711 | H47..00 | Pneumonitis due to inhalation of solids or liquids |
| 38065 | H263.00 | Pneumonitis, unspecified |
| 25462 | A521.00 | Varicella pneumonitis |
| 46066 | H47z.00 | Pneumonitis due to inhalation of solid or liquid NOS |
| 41781 | H470000 | Pneumonitis due to inhalation of regurgitated food |
| 99232 | Hyu4700 | [X]Pneumonitis due to inhalation of other solids and liquids |
| 56762 | AD04.00 | Toxoplasma pneumonitis |
| 47504 | H47y.00 | Pneumonitis due to inhalation of other solid or liquid |
| 103753 | H563.13 | Idiopathic pulmonary fibrosis |
| 6051 | H563100 | Diffuse pulmonary fibrosis |
| 22536 | H4y1000 | Chronic pulmonary fibrosis following radiation |
| Bronchiectasis codes | | |
| 57161 | C390500 | Congenital hypogammaglobulinaemia |
| 2195 | H34..00 | Bronchiectasis |
| 20364 | H340.00 | Recurrent bronchiectasis |
| 32679 | H34z.00 | Bronchiectasis NOS |
| 15137 | C390000 | Hypogammaglobulinaemia NOS |
| 15693 | A115.00 | Tuberculous bronchiectasis |
| 41491 | H341.00 | Post-infective bronchiectasis |
| 92569 | C390800 | Transient infant hypogammaglobulinaemia |
| 56427 | P861.00 | Congenital bronchiectasis |
| Acute Renal Impairment codes | | |
| 31277 | 44J3000 | Serum creatinine abnormal |
| 3927 | 44J3300 | Serum creatinine raised |
| 3980 | 4512.00 | Renal function tests abnormal |
| 2266 | K04..00 | Acute renal failure |
| 105739 | K04..11 | ARF - Acute renal failure |
| 35235 | K04y.00 | Other acute renal failure |
| 25582 | K04z.00 | Acute renal failure NOS |
| 350 | K06..00 | Renal failure unspecified |
| 100205 | K0E..00 | Acute-on-chronic renal failure |
| 53945 | Kyu2000 | [X]Other acute renal failure |
| Chronic Renal Impairment codes | | |
| 12720 | 1Z1..00 | Chronic renal impairment |
| 29013 | 1Z10.00 | Chronic kidney disease stage 1 |
| 12586 | 1Z11.00 | Chronic kidney disease stage 2 |
| 12566 | 1Z12.00 | Chronic kidney disease stage 3 |
| 12479 | 1Z13.00 | Chronic kidney disease stage 4 |
| 12585 | 1Z14.00 | Chronic kidney disease stage 5 |
| 94965 | 1Z15.00 | Chronic kidney disease stage 3A |
| 95179 | 1Z16.00 | Chronic kidney disease stage 3B |
| 94789 | 1Z17.00 | Chronic kidney disease stage 1 with proteinuria |
| 97980 | 1Z17.11 | CKD stage 1 with proteinuria |
| 95572 | 1Z18.00 | Chronic kidney disease stage 1 without proteinuria |
| 95146 | 1Z19.00 | Chronic kidney disease stage 2 with proteinuria |
| 97979 | 1Z19.11 | CKD stage 2 with proteinuria |
| 95121 | 1Z1A.00 | Chronic kidney disease stage 2 without proteinuria |
| 97978 | 1Z1A.11 | CKD stage 2 without proteinuria |
| 94793 | 1Z1B.00 | Chronic kidney disease stage 3 with proteinuria |
| 95145 | 1Z1B.11 | CKD stage 3 with proteinuria |
| 95123 | 1Z1C.00 | Chronic kidney disease stage 3 without proteinuria |
| 95188 | 1Z1C.11 | CKD stage 3 without proteinuria |
| 95408 | 1Z1D.00 | Chronic kidney disease stage 3A with proteinuria |
| 95571 | 1Z1D.11 | CKD stage 3A with proteinuria |
| 95175 | 1Z1E.00 | Chronic kidney disease stage 3A without proteinuria |
| 95176 | 1Z1E.11 | CKD stage 3A without proteinuria |
| 95178 | 1Z1F.00 | Chronic kidney disease stage 3B with proteinuria |
| 95180 | 1Z1F.11 | CKD stage 3B with proteinuria |
| 95177 | 1Z1G.00 | Chronic kidney disease stage 3B without proteinuria |
| 100633 | 1Z1G.11 | CKD stage 3B without proteinuria |
| 95122 | 1Z1H.00 | Chronic kidney disease stage 4 with proteinuria |
| 99312 | 1Z1H.11 | CKD stage 4 with proteinuria |
| 95406 | 1Z1J.00 | Chronic kidney disease stage 4 without proteinuria |
| 97587 | 1Z1J.11 | CKD stage 4 without proteinuria |
| 95508 | 1Z1K.00 | Chronic kidney disease stage 5 with proteinuria |
| 99160 | 1Z1K.11 | CKD stage 5 with proteinuria |
| 95405 | 1Z1L.00 | Chronic kidney disease stage 5 without proteinuria |
| 97683 | 1Z1L.11 | CKD stage 5 without proteinuria |
| 2993 | 4513100 | Creatinine clearance-glom filt abnormal |
| 26001 | 4519.00 | Deteriorating renal function |
| 11773 | 7L1A.11 | Dialysis for renal failure |
| 89332 | 9Ot5.00 | Predicted stage chronic kidney disease |
| 106860 | C353600 | Renal failure-associated hyperphosphataemia |
| 16929 | D215.00 | Anaemia secondary to renal failure |
| 25394 | D215000 | Anaemia secondary to chronic renal failure |
| 32423 | G222.00 | Hypertensive renal disease with renal failure |
| 28684 | G233.00 | Hypertensive heart and renal disease with renal failure |
| 512 | K05..00 | Chronic renal failure |
| 53852 | K05..12 | End stage renal failure |
| 104981 | K05..13 | Chronic kidney disease |
| 6712 | K050.00 | End stage renal failure |
| 105392 | K051.00 | Chronic kidney disease stage 1 |
| 105383 | K052.00 | Chronic kidney disease stage 2 |
| 104619 | K053.00 | Chronic kidney disease stage 3 |
| 104963 | K054.00 | Chronic kidney disease stage 4 |
| 105151 | K055.00 | Chronic kidney disease stage 5 |
| 11787 | K060.00 | Renal impairment |
| 6842 | K060.11 | Impaired renal function |
| 8919 | K08..00 | Impaired renal function disorder |
| 39840 | K08y.00 | Other impaired renal function disorder |
| 50804 | K08yz00 | Other impaired renal function disorder NOS |
| 25980 | K08z.00 | Impaired renal function disorder NOS |
| 8330 | K0D..00 | End-stage renal disease |
| 61930 | Kyu2.00 | [X]Renal failure |
| 53940 | Kyu2100 | [X]Other chronic renal failure |
| 11554 | SP15400 | Renal failure as a complication of care |
| Upper Respiratory Tract infections | | |
| 8980 | 16L..00 | Influenza-like symptoms |
| 98125 | 1J72.00 | Suspected swine influenza |
| 8025 | H0...00 | Acute respiratory infections |
| 3260 | H00..00 | Acute nasopharyngitis |
| 368 | H00..11 | Common cold |
| 1246 | H00..12 | Coryza - acute |
| 6620 | H00..13 | Febrile cold |
| 896 | H00..14 | Nasal catarrh - acute |
| 9093 | H00..15 | Pyrexial cold |
| 3821 | H00..16 | Rhinitis - acute |
| 53395 | H023z00 | Acute bacterial pharyngitis NOS |
| 4868 | H024.00 | Acute viral pharyngitis |
| 407 | H02z.00 | Acute pharyngitis NOS |
| 138 | H03..00 | Acute tonsillitis |
| 11499 | H03..11 | Throat infection - tonsillitis |
| 9357 | H036.00 | Acute viral tonsillitis |
| 20104 | H03z.00 | Acute tonsillitis NOS |
| 41324 | H04..00 | Acute laryngitis and tracheitis |
| 142 | H040.00 | Acute laryngitis |
| 5115 | H040w00 | Acute viral laryngitis unspecified |
| 22720 | H040z00 | Acute laryngitis NOS |
| 1257 | H041.00 | Acute tracheitis |
| 12476 | H041000 | Acute tracheitis without obstruction |
| 68867 | H041100 | Acute tracheitis with obstruction |
| 16313 | H041z00 | Acute tracheitis NOS |
| 10087 | H042.00 | Acute laryngotracheitis |
| 1285 | H042.11 | Laryngotracheitis |
| 25259 | H042000 | Acute laryngotracheitis without obstruction |
| 69898 | H042100 | Acute laryngotracheitis with obstruction |
| 24471 | H042z00 | Acute laryngotracheitis NOS |
| 16120 | H04z.00 | Acute laryngitis and tracheitis NOS |
| 26010 | H05..00 | Other acute upper respiratory infections |
| 18908 | H050.00 | Acute laryngopharyngitis |
| 6294 | H051.00 | Acute upper respiratory tract infection |
| 21415 | H052.00 | Pharyngotracheitis |
| 4221 | H054.00 | Recurrent upper respiratory tract infection |
| 4718 | H055.00 | Pharyngolaryngitis |
| 15628 | H05y.00 | Other upper respiratory infections of multiple sites |
| 76 | H05z.00 | Upper respiratory infection NOS |
| 2637 | H05z.11 | Upper respiratory tract infection NOS |
| 6421 | H05z.12 | Viral upper respiratory tract infection NOS |
| 293 | H06z111 | Respiratory tract infection |
| 21113 | H0z..00 | Acute respiratory infection NOS |
| 556 | H27..00 | Influenza |
| 43625 | H271.00 | Influenza with other respiratory manifestation |
| 15774 | H271000 | Influenza with laryngitis |
| 29617 | H271100 | Influenza with pharyngitis |
| 23488 | H271z00 | Influenza with respiratory manifestations NOS |
| 31363 | H27yz00 | Influenza with other manifestations NOS |
| 16388 | H27z.00 | Influenza NOS |
| 2157 | H27z.11 | Flu like illness |
| 5947 | H27z.12 | Influenza like illness |
| 94930 | H29..00 | Avian influenza |
| 98102 | H2A..11 | Influenza A (H1N1) swine flu |
| 7074 | H5yy.11 | Respiratory infection NOS |
| 53055 | Hyu0.00 | [X]Acute upper respiratory infections |
| 108022 | Hyu0300 | [X]Other acute upper respiratory infections/multiple sites |
| Lower Respiratory Tract infection codes | | |
| 35745 | H270z00 | Influenza with pneumonia NOS |
| 98381 | Hyu0B00 | [X]Pneumonia due to other specified infectious organisms |
| 15912 | H270.00 | Influenza with pneumonia |
| 104315 | A3C0300 | Sepsis due to Streptococcus pneumoniae |
| 17025 | H233.00 | Chlamydial pneumonia |
| 69230 | Q310y00 | Other specified congenital pneumonia |
| 98782 | H24y500 | Pneumonia with toxoplasmosis |
| 23726 | H24y700 | Pneumonia with varicella |
| 5324 | H28..00 | Atypical pneumonia |
| 34300 | H262.00 | Postoperative pneumonia |
| 97634 | Q310000 | Congenital pneumonia due to staphylococcus |
| 104717 | A789311 | HIV disease resulting in Pneumocystis jirovecii pneumonia |
| 106908 | H244.00 | Pneumonia with tularaemia |
| 68 | H06z011 | Chest infection |
| 25694 | H23..00 | Pneumonia due to other specified organisms |
| 9389 | H20..11 | Chest infection - viral pneumonia |
| 60482 | H24y300 | Pneumonia with Q-fever |
| 23546 | H220.00 | Pneumonia due to klebsiella pneumoniae |
| 53969 | H247z00 | Pneumonia with systemic mycosis NOS |
| 31643 | Q310.00 | Congenital pneumonia |
| 49398 | H24y600 | Pneumonia with typhoid fever |
| 60119 | H230.00 | Pneumonia due to Eaton's agent |
| 101204 | H470.11 | Aspiration pneumonia |
| 104121 | H2B..00 | Community acquired pneumonia |
| 27641 | A789300 | HIV disease resulting in Pneumocystis carinii pneumonia |
| 103404 | H247100 | Pneumonia with coccidioidomycosis |
| 4019 | Q310500 | Congenital pneumonia due to viral agent |
| 66362 | H24z.00 | Pneumonia with infectious diseases EC NOS |
| 30653 | H23..11 | Chest infection - pneumonia organism OS |
| 886 | H25..00 | Bronchopneumonia due to unspecified organism |
| 30591 | H221.00 | Pneumonia due to pseudomonas |
| 63763 | Hyu0A00 | [X]Other bacterial pneumonia |
| 6124 | H062.00 | Acute lower respiratory tract infection |
| 31269 | H201.00 | Pneumonia due to respiratory syncytial virus |
| 65419 | H22y000 | Pneumonia due to escherichia coli |
| 12061 | H22y200 | Pneumonia - Legionella |
| 67836 | H200.00 | Pneumonia due to adenovirus |
| 43286 | H241.00 | Pneumonia with cytomegalic inclusion disease |
| 72182 | H24y400 | Pneumonia with salmonellosis |
| 70559 | H24yz00 | Pneumonia with other infectious diseases EC NOS |
| 19400 | H26..11 | Chest infection - pnemonia due to unspecified organism |
| 13573 | H270000 | Influenza with bronchopneumonia |
| 6094 | H2z..00 | Pneumonia or influenza NOS |
| 23333 | H540000 | Hypostatic pneumonia |
| 64799 | H571.00 | Rheumatic pneumonia |
| 62623 | H242.00 | Pneumonia with ornithosis |
| 67901 | H24y100 | Pneumonia with nocardiasis |
| 23298 | 4JRC.00 | Atypical pneumonia screening test |
| 72193 | F00y400 | Meningitis due to klebsiella pneumoniae |
| 5202 | H20..00 | Viral pneumonia |
| 53947 | Hyu0D00 | [X]Pneumonia in viral diseases classified elsewhere |
| 63858 | H223000 | Pneumonia due to streptococcus, group B |
| 103475 | H564.11 | Cryptogenic organising pneumonia |
| 50705 | Q310z00 | Congenital pneumonia NOS |
| 37447 | H06z112 | Acute lower respiratory tract infection |
| 25054 | H470312 | Aspiration pneumonia due to vomit |
| 16287 | H25..11 | Chest infection - unspecified bronchopneumonia |
| 9639 | H260.00 | Lobar pneumonia due to unspecified organism |
| 24356 | H540100 | Hypostatic bronchopneumonia |
| 52071 | H247000 | Pneumonia with candidiasis |
| 23095 | H22z.00 | Bacterial pneumonia NOS |
| 17359 | H30..11 | Chest infection - unspecified bronchitis |
| 52384 | H22yX00 | Pneumonia due to other aerobic gram-negative bacteria |
| 36675 | H202.00 | Pneumonia due to parainfluenza virus |
| 34251 | H23z.00 | Pneumonia due to specified organism NOS |
| 96059 | 4JUK.00 | Mycoplasma pneumoniae detected |
| 4899 | H06z200 | Recurrent chest infection |
| 1576 | H231.00 | Pneumonia due to mycoplasma pneumoniae |
| 1849 | H21..00 | Lobar (pneumococcal) pneumonia |
| 28634 | H22..00 | Other bacterial pneumonia |
| 101292 | AB41500 | Histoplasma duboisii with pneumonia |
| 40299 | AB24.11 | Pneumonia - candidal |
| 37881 | H222.00 | Pneumonia due to haemophilus influenzae |
| 27519 | H24y200 | Pneumonia with pneumocystis carinii |
| 50408 | A730.00 | Ornithosis with pneumonia |
| 68445 | Q310300 | Congenital pneumonia due to Escherichia coli |
| 31024 | A3BXA00 | Mycoplasma pneumoniae [PPLO] cause/dis classifd/oth chaptr |
| 32172 | A551.00 | Postmeasles pneumonia |
| 41034 | H240.00 | Pneumonia with measles |
| 96583 | AyuKA00 | [X]Klebsiella pneumoniae/cause/disease classifd/oth chapters |
| 53753 | Hyu0H00 | [X]Other pneumonia, organism unspecified |
| 62632 | H270100 | Influenza with pneumonia, influenza virus identified |
| 60299 | H22y011 | E.coli pneumonia |
| 29457 | H270.11 | Chest infection - influenza with pneumonia |
| 24316 | H24..11 | Chest infection with infectious disease EC |
| 48982 | Q310200 | Congenital pneumonia due to group B haemolytic streptococcus |
| 33478 | H20y.00 | Viral pneumonia NEC |
| 43884 | H22yz00 | Pneumonia due to bacteria NOS |
| 94165 | Q310600 | Congenital pneumonia due to Chlamydia |
| 3711 | 14B2.00 | H/O: pneumonia |
| 57667 | H530200 | Gangrenous pneumonia |
| 2581 | H06z000 | Chest infection NOS |
| 73735 | H232.00 | Pneumonia due to pleuropneumonia like organisms |
| 45425 | H22y100 | Pneumonia due to proteus |
| 30437 | H243.00 | Pneumonia with whooping cough |
| 61991 | H56y000 | Endogenous lipoid pneumonia |
| 12423 | H223.00 | Pneumonia due to streptococcus |
| 22795 | H22..11 | Chest infection - other bacterial pneumonia |
| 14976 | H20z.00 | Viral pneumonia NOS |
| 99762 | 14B9.00 | History of acute lower respiratory tract infection |
| 35189 | H530300 | Abscess of lung with pneumonia |
| 10086 | H2...00 | Pneumonia and influenza |
| 106031 | AyuK900 | [X]Mycoplasma pneumoniae [PPLO]cause/dis classifd/oth chaptr |
| 22835 | H564.00 | Bronchiolitis obliterans organising pneumonia |
| 98225 | Q310400 | Congenital pneumonia due to pseudomonas |
| 9953 | A116.00 | Tuberculous pneumonia |
| 5612 | H224.00 | Pneumonia due to staphylococcus |
| 106300 | H203.00 | Pneumonia due to human metapneumovirus |
| 31886 | H060A00 | Acute bronchitis due to mycoplasma pneumoniae |
| 54530 | Q310100 | Congenital pneumonia due to group A haemolytic streptococcus |
| 29166 | H21..11 | Chest infection - pneumococcal pneumonia |
| 35082 | H243.11 | Pneumonia with pertussis |
| 40498 | H24..00 | Pneumonia with infectious diseases EC |
| 69782 | H24y.00 | Pneumonia with other infectious diseases EC |
| 61623 | H24y000 | Pneumonia with actinomycosis |
| 50867 | H22y.00 | Pneumonia due to other specified bacteria |
| 34274 | H246.00 | Pneumonia with aspergillosis |
| 26287 | A3BXB00 | Klebsiella pneumoniae/cause/disease classifd/oth chapters |
| 11849 | H2y..00 | Other specified pneumonia or influenza |
| 58896 | A022200 | Salmonella pneumonia |
| 572 | H26..00 | Pneumonia due to unspecified organism |
| 104264 | H2C..00 | Hospital acquired pneumonia |
| 13563 | SP13100 | Other aspiration pneumonia as a complication of care |
| 48804 | H222.11 | Pneumonia due to haemophilus influenzae |
| 41015 | H471000 | Lipoid pneumonia (exogenous) |
| 51398 | A3By400 | Pleuropneumonia-like organism (PPLO) infection |
| 101507 | AB40500 | Histoplasma capsulatum with pneumonia |
| 4910 | H56y100 | Interstitial pneumonia |
| 3683 | H261.00 | Basal pneumonia due to unspecified organism |
| 47973 | A54x400 | Herpes simplex pneumonia |
| 30509 | SP13200 | Post operative chest infection |
| 52520 | Hyu0800 | [X]Other viral pneumonia |
| Sinus infection codes | | |
| 8213 | H011.00 | Acute frontal sinusitis |
| 4433 | H130.00 | Chronic maxillary sinusitis |
| 33664 | H01z.00 | Acute sinusitis NOS |
| 1674 | H132.00 | Chronic ethmoidal sinusitis |
| 97330 | Hyu0000 | [X]Other acute sinusitis |
| 73094 | SN31.11 | Aerosinusitis |
| 5437 | H13z.00 | Chronic sinusitis NOS |
| 2984 | H131.11 | Frontal sinusitis |
| 980 | H01..00 | Acute sinusitis |
| 243 | H01..11 | Sinusitis |
| 15724 | H012.00 | Acute ethmoidal sinusitis |
| 3624 | H130.12 | Maxillary sinusitis |
| 39501 | H13y000 | Chronic pansinusitis |
| 7021 | H010.00 | Acute maxillary sinusitis |
| 49548 | H13y.00 | Other chronic sinusitis |
| 2257 | H13..00 | Chronic sinusitis |
| 48703 | H133.00 | Chronic sphenoidal sinusitis |
| 29696 | H01y.00 | Other acute sinusitis |
| 17173 | H135.00 | Recurrent sinusitis |
| 60733 | H01yz00 | Other acute sinusitis NOS |
| 15163 | H131.00 | Chronic frontal sinusitis |
| 38816 | H013.00 | Acute sphenoidal sinusitis |
| 63733 | Hyu2200 | [X]Other chronic sinusitis |
| 94218 | H014.00 | Acute rhinosinusitis |
| 18572 | H17..12 | Allergic rhinosinusitis |
| 19284 | H01y000 | Acute pansinusitis |
| 10546 | H13..11 | Chronic rhinosinusitis |
| 2233 | H13y100 | Pansinusitis |
| 54375 | H13yz00 | Other chronic sinusitis NOS |
| Urinary Tract Infection codes | | |
| 389 | K15..00 | Cystitis |
| 15074 | K150.00 | Acute cystitis |
| 12484 | K15z.00 | Cystitis NOS |
| 1289 | K190.00 | Urinary tract infection site not specified |
| 97002 | K190500 | Urinary tract infection |
| 150 | K190z00 | Urinary tract infection site not specified NOS |
| Cellulitis codes | |  |
| 4126 | A98yy14 | Gonococcal cellulitis |
| 14937 | M032400 | Cellulitis and abscess of umbilicus |
| 10974 | M036400 | Cellulitis and abscess of ankle |
| 25081 | M02z.00 | Cellulitis and abscess of digit NOS |
| 52366 | M032800 | Cellulitis of trunk |
| 27619 | M034014 | Cellulitis of palm of hand |
| 6833 | M08..00 | Cutaneous cellulitis |
| 48630 | M033z00 | Cellulitis and abscess of arm NOS |
| 20384 | M021z00 | Cellulitis and abscess of toe NOS |
| 30982 | K405100 | Pelvic cellulitis unspecified |
| 7865 | M036.00 | Cellulitis and abscess of leg excluding foot |
| 4456 | K284300 | Cellulitis of scrotum |
| 4748 | J083.00 | Oral cellulitis and abscess |
| 15549 | M030200 | Cellulitis and abscess of chin |
| 5089 | M033000 | Cellulitis and abscess of shoulder |
| 24294 | K404000 | Chronic female pelvic cellulitis |
| 31148 | M037.00 | Cellulitis and abscess of foot excluding toe |
| 29113 | M037z00 | Cellulitis and abscess of foot NOS |
| 8852 | F501112 | Cellulitis, external ear |
| 27757 | M037.11 | Cellulitis and abscess of foot |
| 29345 | M084.00 | [X]Cellulitis of breast |
| 18578 | Q47y200 | Neonatal skin infection |
| 2914 | M034000 | Cellulitis and abscess of hand unspecified |
| 205 | M038.00 | Cellulitis of external ear |
| 21208 | M034013 | Cellulitis of dorsum of hand |
| 1874 | M032200 | Cellulitis and abscess of back |
| 1415 | M034.11 | Cellulitis and abscess of hand |
| 23585 | M032500 | Cellulitis and abscess of flank |
| 16536 | M03..00 | Other cellulitis and abscess |
| 25890 | M036300 | Cellulitis and abscess of lower leg |
| 26239 | K170300 | Periurethral cellulitis |
| 27903 | M033400 | Cellulitis and abscess of forearm |
| 16011 | F4D0.11 | Cellulitis of eyelids |
| 5697 | M02..00 | Cellulitis and abscess of finger and toe |
| 21580 | M030100 | Cellulitis and abscess of nose (external) |
| 1772 | M033100 | Cellulitis and abscess of axilla |
| 4779 | M020.00 | Cellulitis and abscess of finger |
| 14972 | M03y.00 | Other specified cellulitis and abscess |
| 2089 | M037000 | Cellulitis and abscess of foot unspecified |
| 27681 | M030600 | Cellulitis of face |
| 2711 | M031.00 | Cellulitis and abscess of neck |
| 2897 | M035.00 | Cellulitis and abscess of buttock |
| 15687 | K405.00 | Parametritis and pelvic cellulitis unspecified |
| 27908 | M034.00 | Cellulitis and abscess of hand excluding digits |
| 3960 | M021.00 | Cellulitis and abscess of toe |
| 4328 | F4G0100 | Orbital cellulitis |
| 26071 | M020z00 | Cellulitis and abscess of finger NOS |
| 7684 | M08B.00 | Cellulitis of foot |
| 2847 | M036100 | Cellulitis and abscess of thigh |
| 44034 | M033200 | Cellulitis and abscess of upper arm |
| 68242 | F501400 | Infective otitis externa due to erysipelas |
| 20389 | M030z00 | Cellulitis and abscess of face NOS |
| 30260 | M08y.00 | [X]Cellulitis of other sites |
| 27717 | M032.00 | Cellulitis and abscess of trunk |
| 64484 | H1y5100 | Cellulitis of vocal cords |
| 4973 | M032300 | Cellulitis and abscess of abdominal wall |
| 16176 | M032100 | Cellulitis and abscess of breast |
| 2658 | M030011 | Cellulitis and abscess of cheek |
| 36349 | M032z00 | Cellulitis and abscess of trunk NOS |
| 3461 | M033.00 | Cellulitis and abscess of arm |
| 37424 | J540.11 | Perianal cellulitis |
| 17562 | J085100 | Cellulitis of lip |
| 309 | M03z.00 | Cellulitis and abscess NOS |
| 15327 | M030300 | Cellulitis and abscess of submandibular region |
| 15475 | M030400 | Cellulitis and abscess of forehead |
| 17226 | M08A.00 | Cellulitis of axilla |
| 27616 | F501411 | Erysipelas - otitis externa |
| 1156 | A35..00 | Erysipelas |
| 15642 | M037100 | Cellulitis and abscess of heel |
| 16606 | M03..13 | Cellulitis of skin area excluding digits of hand or foot |
| 61518 | H1y7100 | Cellulitis of larynx |
| 4394 | M032000 | Cellulitis and abscess of chest wall |
| 31534 | M086.00 | Cellulitis of ankle |
| 5605 | M080.00 | [X]Cellulitis of finger and toe |
| 24960 | M03y000 | Cellulitis and abscess of head unspecified |
| 3527 | M020000 | Cellulitis and abscess of finger unspecified |
| 7972 | M082.00 | Cellulitis of face |
| 9648 | M088.00 | Cellulitis of arm |
| 16304 | K272300 | Cellulitis of penis |
| 25039 | M083.00 | Cellulitis of trunk |
| 6368 | M085.00 | Cellulitis of leg |
| 19944 | J083z00 | Oral cellulitis and abscess NOS |
| 70783 | K403.00 | Acute parametritis and pelvic cellulitis |
| 7328 | M037200 | Cellulitis in diabetic foot |
| 25156 | H1y2100 | Pharynx or nasopharynx cellulitis |
| 94868 | M08C.00 | Cellulitis of toe |
| 10485 | M030111 | Cellulitis and abscess of nose |
| 23604 | M034z00 | Cellulitis and abscess of hand NOS |
| 1315 | M081.00 | [X]Cellulitis of other parts of limb |
| 28181 | M089.00 | Cellulitis of neck |
| 15336 | J083100 | Oral soft tissue cellulitis unspecified |
| 33659 | K403100 | Acute pelvic cellulitis |
| 2216 | M036200 | Cellulitis and abscess of knee |
| 3998 | M030.00 | Cellulitis and abscess of face |
| 48663 | K404.00 | Chronic parametritis and pelvic cellulitis |
| 9233 | M080.13 | [X]Cellulitis of thumb |
| 4400 | M032700 | Cellulitis and abscess of perineum |
| 16032 | M030500 | Cellulitis and abscess of temple region |
| 680 | M036z00 | Cellulitis and abscess of leg NOS |
| 25403 | M2y7.00 | Eosinophilic cellulitis [Wells] |
| 7821 | F4D1400 | Cellulitis of eyelid |
| 3363 | M021000 | Cellulitis and abscess of toe unspecified |
| 12167 | M03zz00 | Cellulitis and abscess NOS |
| 4207 | M03z000 | Cellulitis NOS |
| 3597 | M036000 | Cellulitis and abscess of hip |
| 10326 | M036.11 | Cellulitis and abscess of leg |
| 3465 | M034100 | Cellulitis and abscess of wrist |
| 3223 | M033300 | Cellulitis and abscess of elbow |
| 1923 | M032600 | Cellulitis and abscess of groin |
| 5228 | J083000 | Cellulitis of floor of mouth |
| 27933 | J54..11 | Cellulitis - anus or rectum |
| 24401 | M030000 | Cellulitis and abscess of cheek (external) |
| Allopurinol codes | |  |
| CPRD prodcode | productname | |
| 46941 | Allopurinol 300mg Tablet (Lagap) | |
| 11975 | Allopurinol 100mg/5ml sugar free oral suspension | |
| 34566 | Allopurinol 300mg tablets (Generics (UK) Ltd) | |
| 34005 | Allopurinol 300mg tablets (Teva UK Ltd) | |
| 52409 | Allopurinol 100mg/5ml oral suspension | |
| 30768 | Allopurinol 100mg tablets (A A H Pharmaceuticals Ltd) | |
| 34930 | Allopurinol 100mg tablets (Wockhardt UK Ltd) | |
| 45352 | Allopurinol 300mg tablets (Ranbaxy (UK) Ltd) | |
| 27535 | ZYLORIC |  |
| 41664 | Allopurinol 100mg Tablet (Celltech Pharma Europe Ltd) | |
| 368 | Zyloric 100mg tablets (Aspen Pharma Trading Ltd) | |
| 54139 | Allopurinol 300mg/5ml oral suspension | |
| 57739 | Allopurinol 100mg/5ml oral solution | |
| 34573 | Allopurinol 300mg tablets (Wockhardt UK Ltd) | |
| 34947 | Allopurinol 100mg Tablet (Lagap) | |
| 41541 | Allopurinol 100mg tablets (Teva UK Ltd) | |
| 34278 | Allopurinol 300mg tablets (A A H Pharmaceuticals Ltd) | |
| 41520 | Allopurinol 300mg tablets (IVAX Pharmaceuticals UK Ltd) | |
| 7805 | Zyloric 300mg tablets (Aspen Pharma Trading Ltd) | |
| 413 | Allopurinol 100mg tablets | |
| 34711 | Allopurinol 100mg tablets (Generics (UK) Ltd) | |
| 33484 | Allopurinol 100mg tablets (Actavis UK Ltd) | |
| 41612 | Allopurinol 300mg tablets (Actavis UK Ltd) | |
| 76 | Allopurinol 300mg tablets | |
| 19201 | Allopurinol 100mg tablets (IVAX Pharmaceuticals UK Ltd) | |
| Carbimazole codes | |  |
| 1715 | Carbimazole 20mg tablets | |
| 57640 | Carbimazole 20mg tablets (A A H Pharmaceuticals Ltd) | |
| 350 | Carbimazole 5mg tablets | |
| 24686 | CARBIMAZOLE | |
| Levothyroxine codes | | |
| 61437 | Levothyroxine sodium 25microgram capsules | |
| 37906 | Levothyroxine sodium 250micrograms/5ml oral suspension | |
| 59237 | Levothyroxine sodium 100micrograms/5ml oral solution | |
| 40244 | Levothyroxine sodium 100microgram tablets (Almus Pharmaceuticals Ltd) | |
| 41413 | Levothyroxine sodium 50microgram tablets (Almus Pharmaceuticals Ltd) | |
| 54636 | Levothyroxine sodium 37.5micrograms/5ml oral suspension | |
| 59837 | Levothyroxine sodium 100micrograms/5ml oral suspension | |
| 37863 | Levothyroxine 25microgram Tablet (Goldshield Pharmaceuticals Ltd) | |
| 32075 | Levothyroxine sodium 100microgram tablets (Generics (UK) Ltd) | |
| 54370 | Levothyroxine sodium 50micrograms/5ml oral suspension | |
| 54303 | Levothyroxine sodium 200microgram powder for solution for injection vials | |
| 57685 | Levothyroxine sodium 500microgram powder for solution for injection vials | |
| 13370 | ELTROXIN 25 MCG TAB | |
| 6494 | Levothyroxine lactose free Oral suspension | |
| 53083 | Levothyroxine sodium 9.5microgram / Liothyronine 2.25microgram tablets | |
| 25082 | ELTROXIN | |
| 60860 | Levothyroxine sodium 19microgram / Liothyronine 4.5microgram tablets | |
| 27 | Levothyroxine sodium 25microgram tablets | |
| 29466 | Levothyroxine sodium 100microgram tablets (Actavis UK Ltd) | |
| 30271 | Levothyroxine sodium 50micrograms/5ml oral solution sugar free | |
| 10 | Levothyroxine sodium 100microgram tablets | |
| 48454 | Eltroxin 25micrograms/5ml oral solution (AMCo) | |
| 46928 | Levothyroxine 100microgram Tablet (C P Pharmaceuticals Ltd) | |
| 53764 | Levothyroxine sodium 25microgram tablets (AMCo) | |
| 18198 | Levothyroxine 50micrograms/5ml Oral suspension | |
| 51477 | Levothyroxine sodium 57microgram / Liothyronine 13.5microgram tablets | |
| 45270 | Levothyroxine Capsule | |
| 60037 | Levothyroxine sodium 25microgram tablets (DE Pharmaceuticals) | |
| 27266 | Levothyroxine sodium 25microgram tablets (A A H Pharmaceuticals Ltd) | |
| 33976 | Levothyroxine sodium 25microgram tablets (Teva UK Ltd) | |
| 25567 | Levothyroxine sodium 100microgram tablets (IVAX Pharmaceuticals UK Ltd) | |
| 25566 | Levothyroxine sodium 25microgram tablets (IVAX Pharmaceuticals UK Ltd) | |
| 29182 | Levothyroxine sodium 100micrograms/5ml oral solution sugar free | |
| 53184 | Levothyroxine Oral suspension | |
| 53683 | Levothyroxine sodium 125micrograms/5ml oral solution | |
| 57857 | Levothyroxine sodium 76microgram / Liothyronine 18microgram tablets | |
| 58245 | Levothyroxine sodium 100microgram capsules | |
| 3941 | Eltroxin 50microgram tablets (AMCo) | |
| 53167 | Levothyroxine sodium 50micrograms/5ml oral solution sugar free (A A H Pharmaceuticals Ltd) | |
| 32073 | Levothyroxine sodium 50microgram tablets (Generics (UK) Ltd) | |
| 37844 | Levothyroxine sodium 250micrograms/5ml oral solution | |
| 51487 | Eltroxin 100micrograms/5ml oral solution (AMCo) | |
| 32072 | Levothyroxine sodium 25microgram tablets (Generics (UK) Ltd) | |
| 60505 | Levothyroxine sodium 50micrograms/5ml oral solution | |
| 32915 | Levothyroxine sodium 50microgram tablets (Actavis UK Ltd) | |
| 51718 | Levothyroxine sodium 100microgram tablets (AMCo) | |
| 13 | Levothyroxine sodium 50microgram tablets | |
| 51717 | Levothyroxine sodium 50microgram tablets (AMCo) | |
| 3940 | Eltroxin 100microgram tablets (AMCo) | |
| 13973 | Eltroxin 25microgram tablets (AMCo) | |
| 57394 | Levothyroxine sodium 100microgram oral powder sachets | |
| 42219 | Levothyroxine sodium 100micrograms/5ml oral solution sugar free (A A H Pharmaceuticals Ltd) | |
| 53266 | Levothyroxine sodium 125micrograms/5ml oral suspension | |
| 27688 | Levothyroxine sodium 25microgram tablets (Actavis UK Ltd) | |
| 50345 | Eltroxin 50micrograms/5ml oral solution (AMCo) | |
| 29660 | Levothyroxine sodium 50microgram tablets (IVAX Pharmaceuticals UK Ltd) | |
| 10132 | Levothyroxine lactose free Oral suspension | |
| 11857 | Levothyroxine 25micrograms/5ml Oral suspension | |
| 34220 | Levothyroxine lactose free 100microgram/5ml Oral suspension (Martindale Pharmaceuticals Ltd) | |
| 59541 | Levothyroxine sodium 50microgram capsules | |
| 34402 | Levothyroxine sodium 50microgram tablets (A A H Pharmaceuticals Ltd) | |
| 24994 | Levothyroxine sodium 25micrograms/5ml oral solution sugar free | |
| 47318 | Levothyroxine sodium 25microgram capsules | |
| 45238 | Levothyroxine 50microgram Tablet (Sigma Pharmaceuticals Plc) | |
| 33084 | Levothyroxine sodium 100microgram tablets (Teva UK Ltd) | |
| 41519 | Levothyroxine sodium 25microgram tablets (Wockhardt UK Ltd) | |
| 50190 | Levothyroxine sodium 75microgram capsules | |
| 58907 | Levothyroxine sodium 25micrograms/5ml oral suspension | |
| 58538 | Levothyroxine sodium 38microgram / Liothyronine 9microgram tablets | |
| 43739 | Levothyroxine lactose free 25microgram/ml Oral suspension (Martindale Pharmaceuticals Ltd) | |
| 34420 | Levothyroxine sodium 100microgram tablets (A A H Pharmaceuticals Ltd) | |
| 27448 | Levothyroxine sodium 50microgram tablets (Teva UK Ltd) | |
| 56057 | Levothyroxine sodium 25micrograms/5ml oral solution sugar free (A A H Pharmaceuticals Ltd) | |
| Propylthiouracil codes | | |
| 1365 | PROPYLTHIOURACIL 150 MG TAB | |
| 34723 | Propylthiouracil 50mg Tablet (UCB Pharma Ltd) | |
| 673 | Propylthiouracil 50mg tablets | |
| 30901 | PROPYLTHIOURACIL 500 MG TAB | |
| 10855 | PROPYLTHIOURACIL BP 100 MG TAB | |
| 17089 | PROPYLTHIOURACIL 25 MG TAB | |
| 55213 | Propylthiouracil 50mg tablets (A A H Pharmaceuticals Ltd) | |
| Hydralazine codes | |  |
| 31971 | HYDRALAZINE 6.25 MG SYR | |
| 31220 | Hydralazine 25mg tablets (A A H Pharmaceuticals Ltd) | |
| 21749 | HYDRALAZINE HCl 100 MG TAB | |
| 2680 | Apresoline 50mg Tablet (Sovereign Medical Ltd) | |
| 4507 | HYDRALAZINE HCl 12.5 MG TAB | |
| 41639 | Hydralazine 50mg tablets (Actavis UK Ltd) | |
| 504 | Hydralazine 20mg powder for solution for injection ampoules | |
| 23746 | HYDRALAZINE HCl 10 MG TAB | |
| 13317 | Apresoline 20mg powder for solution for injection ampoules (AMCo) | |
| 18861 | Hydralazine 10mg/5ml oral suspension | |
| 59512 | Hydralazine 50mg/5ml oral solution | |
| 61116 | Hydralazine 50mg/5ml oral suspension | |
| 43500 | Hydralazine 25mg tablets (Actavis UK Ltd) | |
| 1296 | Hydralazine 50mg tablets | |
| 573 | Hydralazine 25mg tablets | |
| 2362 | Apresoline 25mg tablets (AMCo) | |
| 214 | HYDRALAZINE 1 MG SYR | |
| Minocycline codes | |  |
| 1532 | Minocycline 100mg tablets | |
| 40383 | Minocycline 100mg tablets (Actavis UK Ltd) | |
| 9380 | Minocycline 100mg capsules | |
| 21978 | Blemix 100mg tablets (Ashbourne Pharmaceuticals Ltd) | |
| 46947 | Minocycline 50mg tablets (Actavis UK Ltd) | |
| 14984 | Acnamino MR 100mg capsules (Dexcel-Pharma Ltd) | |
| 29418 | Dentomycin 2% w/v Dental gel (Wyeth Pharmaceuticals) | |
| 18684 | Aknemin 50 capsules (Almirall Ltd) | |
| 32588 | Dentomycin 2% dental gel (Henry Schein Ltd) | |
| 54152 | Acnamino MR 100mg capsules (Almus Pharmaceuticals Ltd) | |
| 46954 | Minocycline 100mg Tablet (Lagap) | |
| 2578 | Minocycline 100mg modified-release capsules | |
| 34077 | Minocycline 100mg modified-release capsules (A A H Pharmaceuticals Ltd) | |
| 1039 | Minocin MR 100mg capsules (Meda Pharmaceuticals Ltd) | |
| 34926 | Minocycline 100mg tablets (A A H Pharmaceuticals Ltd) | |
| 24245 | Cyclomin 100mg Tablet (Berk Pharmaceuticals Ltd) | |
| 2999 | Minocycline 50mg tablets | |
| 3413 | Minocin 100mg tablets (Wyeth Pharmaceuticals) | |
| 43700 | Minocycline 50mg Tablet (Lagap) | |
| 18728 | Aknemin 100mg capsules (Almirall Ltd) | |
| 164 | Minocin 50mg tablets (Wyeth Pharmaceuticals) | |
| 18109 | Sebomin MR 100mg capsules (Actavis UK Ltd) | |
| 37440 | Sebren MR 100mg capsules (Teva UK Ltd) | |
| 17705 | Minocycline 2% dental gel sugar free | |
| 429 | Minocycline 50mg capsules | |
| 59039 | Minocycline 100mg tablets (Generics (UK) Ltd) | |
| 59922 | Minocycline 100mg tablets (Teva UK Ltd) | |
| 21837 | Cyclomin 50mg Tablet (Berk Pharmaceuticals Ltd) | |
| 21865 | Blemix 50mg tablets (Ashbourne Pharmaceuticals Ltd) | |
| Sulphasalzine codes | | |
| 55395 | Sulfasalazine 500mg tablets (Waymade Healthcare Plc) | |
| 56909 | Sulfasalazine 500mg tablets (Phoenix Healthcare Distribution Ltd) | |
| 33968 | Sulfasalazine 500mg Tablet (Approved Prescription Services Ltd) | |
| 58671 | Sulfasalazine 500mg tablets (Almus Pharmaceuticals Ltd) | |
| 15373 | Sulfasalazine 500mg suppositories | |
| 34473 | Sulfasalazine 500mg tablets (Generics (UK) Ltd) | |
| 31683 | Sulfasalazine 3g/100ml retention enema | |
| 33682 | Sulfasalazine 500mg Gastro-resistant tablet (DDSA Pharmaceuticals Ltd) | |
| 2920 | Sulfasalazine 500mg tablet | |
| 61084 | Sulfasalazine 500mg gastro-resistant tablets (Kent Pharmaceuticals Ltd) | |
| 7497 | Sulfasalazine 250mg/5ml oral solution | |
| 31949 | Sulfasalazine 500mg tablets (Actavis UK Ltd) | |
| 59099 | Sulfasalazine 500mg tablets (Alliance Healthcare (Distribution) Ltd) | |
| 5427 | Sulfasalazine 500mg tablets | |
| 49244 | Sulfasalazine 250mg/5ml oral suspension sugar free | |
| 34894 | Sulfasalazine 500mg Gastro-resistant tablet (Ceretron Ltd) | |
| 42178 | Sulfasalazine 500mg Tablet (Berk Pharmaceuticals Ltd) | |
| 508 | Sulfasalazine 500mg gastro-resistant tablets | |
| 20862 | Sulfasalazine 500mg gastro-resistant tablets (Actavis UK Ltd) | |
| 31667 | Sulfasalazine 500mg tablets (A A H Pharmaceuticals Ltd) | |
| 11767 | Sulfasalazine 250mg/5ml oral suspension | |
| 60606 | Sulfasalazine 500mg gastro-resistant tablets (Sigma Pharmaceuticals Plc) | |
| 44183 | Sulfasalazine 500mg gastro-resistant tablets (A A H Pharmaceuticals Ltd) | |
| 359 | Sulfasalazine 3g/100ml enema | |
| 49243 | Sulfasalazine 500mg/5ml oral suspension | |
| 3697 | Sulfasalazine 500mg suppositories | |
| 54209 | Sulfasalazine 500mg gastro-resistant tablets (Phoenix Healthcare Distribution Ltd) | |
| Penicillamine codes | | |
| 57183 | Penicillamine 125mg tablets (A A H Pharmaceuticals Ltd) | |
| 29721 | Pendramine 125mg Tablet (Viatris Pharmaceuticals Ltd) | |
| 28199 | PENICILLAMINE 500 MG SUS | |
| 56872 | Penicillamine 125mg tablets (Phoenix Healthcare Distribution Ltd) | |
| 40170 | Penicillamine 125mg tablets (Generics (UK) Ltd) | |
| 11959 | Distamine 50mg Tablet (Alliance Pharmaceuticals Ltd) | |
| 20255 | Pendramine 250mg Tablet (Viatris Pharmaceuticals Ltd) | |
| 59312 | Penicillamine 125mg tablets (Kent Pharmaceuticals Ltd) | |
| 31216 | Penicillamine 125mg tablets (Actavis UK Ltd) | |
| 31217 | Penicillamine 250mg tablets (A A H Pharmaceuticals Ltd) | |
| 31120 | Penicillamine 125mg Tablet (IVAX Pharmaceuticals UK Ltd) | |
| 8904 | Distamine 250mg tablets (Alliance Pharmaceuticals Ltd) | |
| 30925 | Penicillamine 250mg tablets (Actavis UK Ltd) | |
| 34684 | Penicillamine 250mg tablets (Generics (UK) Ltd) | |
| 48061 | Penicillamine oral liquid | |
| 57789 | Penicillamine 125mg tablets (Alliance Healthcare (Distribution) Ltd) | |
| 60689 | Penicillamine 125mg/5ml oral solution | |
| 54257 | Penicillamine 250mg tablets (Alliance Healthcare (Distribution) Ltd) | |
| 604 | Penicillamine 250mg tablets | |
| 352 | PENICILLAMINE 125 MG CAP | |
| 3327 | Distamine 125mg tablets (Alliance Pharmaceuticals Ltd) | |
| 8392 | PENICILLAMINE 250 MG CAP | |
| 55433 | Penicillamine 250mg tablets (Kent Pharmaceuticals Ltd) | |
| 58953 | Penicillamine 250mg tablets (Waymade Healthcare Plc) | |
| 267 | Penicillamine 50mg tablets | |
| 58468 | Penicillamine 125mg tablets (Teva UK Ltd) | |
| 643 | Penicillamine 125mg tablets | |
| 52868 | Penicillamine 250mg tablets (Phoenix Healthcare Distribution Ltd) | |
